# Marine Anthraquinones: Pharmacological and Toxicological Issues

**DOI:** 10.3390/md19050272

**Published:** 2021-05-13

**Authors:** Giulia Greco, Eleonora Turrini, Elena Catanzaro, Carmela Fimognari

**Affiliations:** Department for Life Quality Studies, Alma Mater Studiorum, Università di Bologna, Corso d’Augusto 237, 47921 Rimini, Italy; giulia.greco9@unibo.it (G.G.); eleonora.turrini@unibo.it (E.T.); elena.catanzaro2@unibo.it (E.C.)

**Keywords:** anthraquinones, marine organisms, fungi, in vitro studies, in vivo studies, cytotoxicity, anticancer mechanisms, genotoxicity

## Abstract

The marine ecosystem, populated by a myriad of animals, plants, and microorganisms, is an inexhaustible reservoir of pharmacologically active molecules. Among the multiple secondary metabolites produced by marine sources, there are anthraquinones and their derivatives. Besides being mainly known to be produced by terrestrial species, even marine organisms and the uncountable kingdom of marine microorganisms biosynthesize anthraquinones. Anthraquinones possess many different biological activities, including a remarkable antitumor activity. However, due to their peculiar chemical structures, anthraquinones are often associated with toxicological issues, even relevant, such as genotoxicity and mutagenicity. The aim of this review is to critically describe the anticancer potential of anthraquinones derived from marine sources and their genotoxic and mutagenic potential. Marine-derived anthraquinones show a promising anticancer potential, although clinical studies are missing. Additionally, an in-depth investigation of their toxicological profile is needed before advocating anthraquinones as a therapeutic armamentarium in the oncological area.

## 1. Introduction

Cancer continues to be a harmful enemy to human health worldwide. If in 2020 the cancer cases were about 20 million, it is estimated that this number can more or less double in 2040 [1]. Thus, a continuous and increasingly efficient effort is needed to fight the burden of cancer.

**Figure 1:**
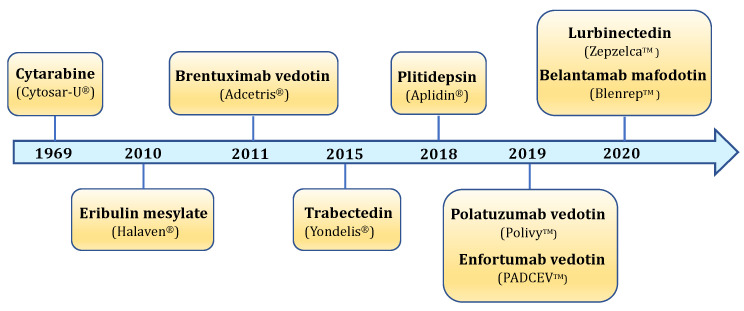
Timeline describing all the marine-derived agents approved as anticancer drugs.

The marine ecosystem is characterized by an incredible biodiversity. It is composed of thousands of different animal and plant species, each of which can produce an infinitely large number of biologically active molecules. Indeed, it is estimated that over 10 million species of prokaryotic and eukaryotic organisms could inhabit the marine environment, but only 250,000 have been currently described [2]. The enormous biodiversity of marine organisms results in a chemical variety of molecules produced as secondary metabolites. Marine secondary metabolites are produced in order to survive or adapt to extreme environmental conditions, and to defense against possible predators or pathogenic organisms. Besides their protective role, secondary metabolites possess many biological effects. Indeed, all over the world, marine flora and fauna have been used for medical uses since ancient times [3]. Nowadays, the impressive potential of marine natural products is increasingly recognized, particularly in the oncological field. Between 2018 and the end of 2020, five new marine-derived drugs have been approved (plitidepsin, polatuzumab vedotin, enfortumab vedotin, belantamab mafodotin, and lurbinectedin) in addition to the four anticancer drugs already approved before 2018 (cytarabine, trabectedin, eribulin mesylate, and the antibody–drug conjugate (ADC) brentuximab vedotin). Hence, nine marine-derived drugs are currently available as anticancer chemotherapeutics (Figure 1) [4]. In addition, a great number of marine-derived compounds are actually in preclinical investigations, while 19 are being tested in different stages of clinical trials [4].

**Figure 2:**
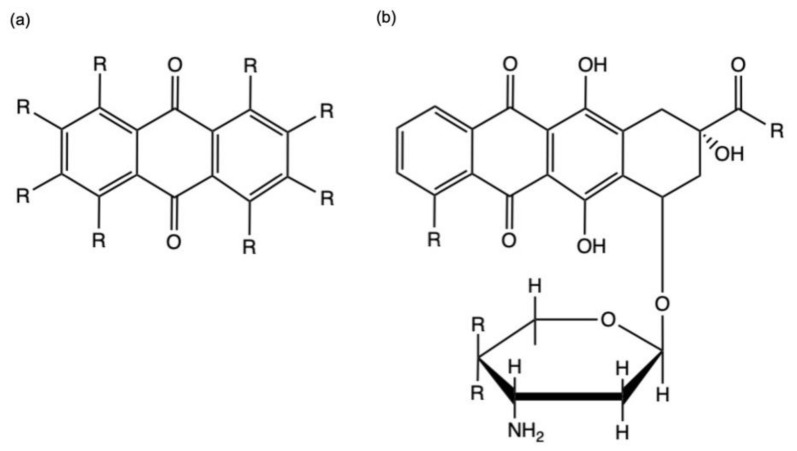
Chemical structure of anthraquinones’ (**a**) and anthracyclines’ core (**b**).

Anthraquinones (AQs) are a class of phenolic compounds characterized by a 9,10-anthracenedione (also called 9,10-dioxoanthracene) core structure composed by three fuse benzene rings with two ketone groups on the central rings (Figure 2a). AQs could be isolated either as glycosides (i.e., linked to a sugar residue) or as their free and pharmacologically active form aglycones [5]. For example, anthracyclines, a class of antitumor agents belong to anthraquinoids’ family, are composed of an anthraquinone tricyclic ring system linked to an amino-sugar moiety (Figure 2b) [6].

To date, about 700 molecules related to AQs have been described. Of these, approximately 200 AQs were isolated from plants, while the remaining were obtained from other terrestrial and marine sources, such as fungi, bacteria, lichens, sponges, and marine invertebrates [7]. Furthermore, the same AQ derivatives could be found in both terrestrial and marine environment. For example, the most known and characterized AQs emodin, physcion, and chrysophanol were isolated from terrestrial sources (plants and endophytic fungi), but also from marine fungi as those belonging to *Aspergillus*, *Penicillium*, and *Microsporum* sp. [5].

AQs and their derivatives are increasingly attracting attention, thanks to their multiple biological activities, such as laxative [8], antifungal [9], antibacterial [10], antimalarial [11], anti-inflammatory [12,13], antiarthritic [13], diuretic [12], antiplatelet [14,15], neuroprotective [16], and anticancer activity [5,8,17,18,19]. Thus far, six natural and semi-synthetic anthracyclines were approved by the FDA (Food and Drug Administration) as anticancer drugs: daunorubicin, adriamycin, idarubicin, epirubicin, valrubicin, and mitoxantrone [8].

The aim of this review is to outline the potential antitumor activity of marine-derived AQs. In vitro and in vivo studies related to AQs isolated from marine sources are critically described and discussed. Additionally, we highlight their genotoxic and mutagenic activity, which today is the subject of a heated debate in Europe.

## 2. Anticancer Mechanisms of AQs Isolated from Marine Fungi

A definition for marine fungi that is accepted by all mycologists is still a matter of debate. In 1979, Kohlmeyer et al. [20] suggested a wide ecological definition, used for over 35 years, of marine fungi, subdividing them into two classes: obligate and facultative marine fungi. Obligate marine fungi grow and sporulate exclusively in seawater; facultative marine fungi, instead, derive from terrestrial environment or freshwater but developed the ability to survive, grow, and sporulate in marine habitats [20]. More recently, Pang and colleagues [21] attempted to give a more comprehensive definition of marine fungus defining it as ‘any fungus that is recovered repeatedly from marine habitats because: (1) it is able to grow and/or sporulate (on substrata) in marine environments; (2) it forms symbiotic relationships with other marine organisms; or (3) it is shown to adapt and evolve at the genetic level or be metabolically active in marine environments’ [21].

Nowadays, fungi are identified as an inexhaustible reservoir of bioactive secondary metabolites [22,23,24], including many different AQ derivatives. Besides conferring unique colors to different fungal structures as spores, sexual bodies, and sclerotia, AQs play an important biological role in fungi: they are produced in order to increase fungi resistance to a range of environmental detrimental factors [5].

The biosynthesis of AQs in fungi and plants is different. In fungi, polyketide compounds, such as AQs, are mainly synthesized by the acetate–malonate pathway, while in plants they are produced through the shikimate and acetate–malonate pathways [25]. In fungi, AQs biosynthesis is regulated by non-reducing polyketide synthases (NR-PKSs) enzymes, which control the regioselective cyclization of the β-polyketide chain, thus dictating the final aromatic structures. These multidomain enzyme complexes catalyze the condensation of an acetyl-CoA molecule (starter unit) to a malonyl-CoA molecule (extender unit) and produce an instable β-polyketide chain containing a free carboxylate group, which is the precursor of many different AQs [25]. The biosynthesis of secondary metabolites in fungi varies depending on multiple factors such as the presence of acetate, malonate or both elements, the fungi’s growth conditions, the fungal strains involved, and the number of each incorporated residue [5,25].

Several AQs isolated from marine fungi display cytotoxic effects towards multiple in vitro cancer cell models, as described in Table 1. The anticancer mechanisms underpinning the cytotoxicity of AQs from marine-derived fungi are poorly characterized. Thus, herein we describe the few marine AQs with well-defined antitumor mechanisms.

### 2.1. Emodin

**Figure 3:**
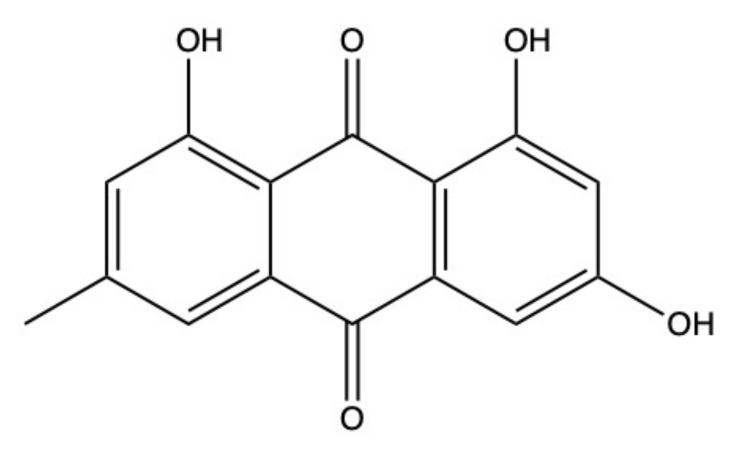
Chemical structure of emodin.

Emodin (Figure 3) is definitely one of the most known and characterized AQs. A great number of studies unraveled emodin’s antitumor potential of both terrestrial and marine extraction, which are factually the same molecule [68]. The amount of information regarding this compound could be used to write an entire article. Thus, in compliance with the review’s main aim, which is to describe the anticancer potential of the most important AQs derived from marine sources, we will focus on the anticancer activity of emodin isolated only from the aquatic environment. For a more comprehensive analysis regarding emodin, we refer to Tuli and colleagues’ review, which is univocally focused on the characterization of emodin’s therapeutic potential [68].

Emodin is isolated from different marine-derived fungal species, such as the coral-derived *Aspergillus tritici* and the sponge-derived *Penicillium* sp. and *Neosartorya fischeri* [28,55,56]. Marine-derived emodin was tested on different solid cancer models. In gastric MGC803 cancer cells, emodin showed a much stronger cytotoxicity (concentration reducing cell viability by 50% (IC_50_): 5.19 μM)) [56] compared to other solid tumors (i.e., ovarian, lung, and hepatocellular), where the IC_50_ values were almost 4- to 6-fold higher [28] (Table 1). However, Tan and colleagues found that in another gastric cell line (SCG7901) emodin 200 μg/mL (740 μM) decreased cell proliferation of only 32.7% after 72 h, thus showing much lesser activity [55]. This discrepancy could be attributed to two different factors: 1) the different method used to evaluate the cytotoxic or antiproliferative effect and 2) the fact that SCG7901 cells are known to be contaminated with human cervical carcinoma HeLa cells [69,70]. More in detail, an MTT assay, based on the measure of the mitochondrial dehydrogenase activity [71], was used to test emodin in SCG7901 cells, while the most sensitive Cell Counting Kit 8 (CCK-8) assay, based on the measure of all dehydrogenase activity of viable cells [71], was used to test emodin cytotoxic effects in MGC803 cells. In addition, for the reason mentioned above, SCG7901 cells should be considered as a human papillomavirus-related endocervical adenocarcinoma cell line rather than a gastric cell line. Therefore, it is much more likely that the different cytotoxic activity of emodin is due to a different sensitivity of the two tumor types.

Generally, the antitumor potential of emodin relies upon multiple mechanisms, including the ability to induce apoptosis. In mitochondrial (or intrinsic) apoptosis, the collapse of the transmembrane potential promotes the cytoplasmatic release of multiple apoptogenic factors, such as cytochrome *c* [72]. Then, the formation of the so-called complex apoptosome leads to the recruitment and activation of pro-caspase-9, which consequently catalyzes the proteolytic activation of executioner caspase-3 and -7, driving to apoptosis execution by cleaving different nuclear substrates such as PARP (poly-(ADP-ribose)-polymerase) [72]. In the death receptor (or extrinsic) apoptotic pathway, instead, activated specific transmembrane death receptors (DRs) bind to their corresponding adaptive cytoplasmic proteins and pro-caspase-8 or -10, leading to the development of the so-called death-inducing signaling complex (DISC). Then, DISC promotes the auto-catalytic activation of pro-caspase-8/-10, which in turn cleave and activate effector caspase-3/-7, leading to apoptotic cell death [73]. Emodin activates intrinsic, extrinsic, or both apoptotic pathways in multiple types of cancer [68]. As an example, in lung, ovarian, cervical, and leukemia cancer cells, emodin seems to preferentially activate mitochondrial apoptosis, upon the increase in ROS (reactive oxygen species) generation and the perturbation of apoptosis-related proteins, which converge to the cleavage of caspase-9 and consequently the cleavage of caspase-3, and its substrate PARP [18].

PI3K (phosphoinositide 3-kinase)/Akt (protein kinase B)/mTOR (mammalian target of rapamycin) signaling pathway is involved in many different biological processes, such as cell proliferation and apoptosis. Activation of PI3K promotes the phosphorylation of different downstream target molecules as Akt and mTOR [74]. Once Akt is activated, it could phosphorylate multiple target proteins, including GSK-3 (glycogen synthase kinase-3), FoxOs (forkhead box O), Bad (Bcl-2 associated agonist of cell death), caspase-9, NF-kB (nuclear transcription factor-kappa B), mTOR, and p21 (cyclin-dependent kinase inhibitor 1A), thus modulating cell growth, proliferation, apoptosis, cell cycle, and glucose metabolism [74]. Dysregulation of this molecular pathway, particularly its aberrant activation, could promote tumor development and progression, thus contributing to chemoresistance and increased tumor aggressiveness [75]. For this reason, the inhibition of the PI3K/Akt/mTOR signaling pathway is considered an important target in cancer treatment. In acute myeloid leukemia (AML) cells, emodin-induced apoptosis was associated with the suppression of the PI3K/Akt/mTOR cell signaling. In particular, emodin efficiently downregulated Akt and mTOR phosphorylation, thus suppressing the effect of their downstream targets [5]. It is worth noting that different marine AQs shared with emodin the ability to inhibit this molecular pathway, as will be discussed later.

To date, it is well known that a wide variety of natural products could promote non-apoptotic (or non-canonical) programmed cell death mechanisms, which are often caspase-independent, including for example necroptosis and ferroptosis [76]. If emodin’s ability to induce apoptosis has been known for several years, its ability to induce non-canonical cell death is much more recent. In fact, only last year Zhou and colleagues found that emodin (20 μM) promoted necroptosis in human glioma U251 cells through the activation of RIP (receptor interacting protein) 1 and RIP3, which are the two main molecular actors in necroptotic cell death [77]. Induction of necroptosis by emodin was also confirmed in U251 xenografted BALB/c-nu/nu nude mice intragastrically treated with 20, 40, or 80 mg emodin/kg body weight (bw)/day for 4 weeks. Emodin significantly reduced tumor growth promoting necroptosis, as demonstrated by the increased levels of TNF-α (tumor necrosis factor α), RIP1, RIP3, and MLKL (mixed lineage kinase domain-like protein) observed in tumor tissues of the emodin-treated group [77].

Emodin, together with other marine AQs, triggers anticancer mechanisms through the post-transcriptional regulation of different miRNAs. miRNAs are a family of small non-coding RNAs that control multiple biological and pathological processes, including cell proliferation, differentiation, apoptosis, and cell cycle. Additionally, they regulate the response of cancer cells to drugs and the development of chemoresistance [78]. miRNA expression is dysregulated in different solid and hematopoietic cancers, where they act as oncogene and/or tumor suppressor genes in a tumor type-dependent fashion [78]. For example, physiologically, miRNA-1271 acts as tumor suppressor by inhibiting epithelial mesenchymal transition (EMT). Emodin inhibited cell proliferation, EMT, and invasion of pancreatic tumor cells (SW1990) in vitro by the upregulation of miRNA-1271 [79]. The anticancer effects of emodin mediated by miRNA-1271 were confirmed in nude mice (strain was not indicated) inoculated with SW1990 cells and treated (oral gavage) with 20 or 50 mg emodin/kg bw per day [79]. The hepatic metastasis of pancreatic carcinoma recorded in emodin-treated mice were significantly lower than in the control group [79]. Moreover, emodin (20, 40, or 80 mg/kg, 3 times a week for 2 weeks) suppressed angiogenesis in an orthotopically transplanted pancreatic cancer model by modulating the angiogenesis-associated miRNA-155, miRNA-210, and miRNA-20b [80]. In particular, RT-qPCR performed on pancreatic tumor tissues unveiled that emodin upregulated miRNA-20b expression and downregulated miRNA-155 and miRNA-210 expression [80].

In addition to the aforementioned anticancer mechanisms, emodin displayed antimutagenic activity, meaning that it is able to lessen or abolish mutation occurrence induced by mutagens. Lee and Tsai found that emodin (1.6–50 μg/plate) strongly inhibited the mutagenicity of 2-amino-3-methylimidazo[4,5-f]quinoline (IQ), 3-amino-t-methyl-5H-pyrido[4,3-b]indole (Trp-P-2), and benzo[a]pyrene (B[a]P) (IQ > Trp-P-2 > B[a]P) in the Ames test with the *Salmonella typhimurium* TA98 strain and S9 mix as a metabolic activation system [81]. In the same experimental system, Su et al. demonstrated that emodin dose-dependently (6.25–100 μg/plate) inhibited the mutagenicity of 1-nitropyrene (1-NP) [82]. Moreover, emodin (1–100 μM) also dose-dependently inhibited B[a]P-induced DNA damage in hepatocellular HepG2 cancer cells [83]. Antimutagenic agents are classified into two main categories: desmutagens and bio-antimutagens. Desmutagens inactivate mutagens before reaching their cellular targets. Bio-antimutagens, instead, act after DNA damage, promoting DNA repair or preventing DNA replication [84]. Antimutagenesis involves different mechanisms, including chemical or enzymatic inactivation of mutagens through the modulation of phase 1 and phase 2 enzymes, prevention of mutagen’s formation, free radical scavenging activity, and antioxidant activity [85,86]. Different studies pointed out that emodin could be classified as a desmutagen agent. In particular, emodin suppressed IQ mutagenicity through the inhibition of hepatic microsomal activity [81], while the inhibition of the N-hydroxylation activity of cytochrome P450 (CYP) 1A1 by emodin was responsible for the suppression of Trp-P-2-induced DNA damage [87]. Additionally, emodin inhibited the mutagenicity of 1-NP by blocking the formation of 8-substituted deoxyguanosine DNA adducts, which is due to the suppression of the nitroreductase enzymatic activity [82].

Emodin’s antimutagenic activity could be attributed to its radical scavenger activity. Indeed, Sevcovicova et al. [88] associated the ability of emodin to protect plasmid DNA from Fe^2+^-induced DNA damage with its hydroxyl radical scavenging activity [88].

Taken together, the antimutagenic potential of emodin relies on its ability to inhibit mutagens’ metabolic activation and quenching oxidative stress.

### 2.2. Physcion

**Figure 4:**
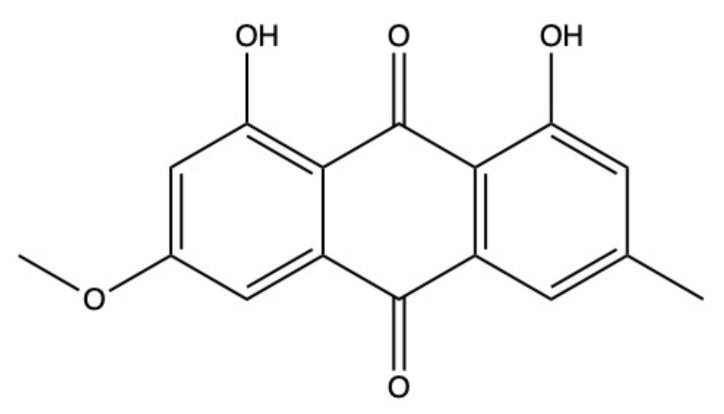
Chemical structure of physcion.

Physcion (Figure 4) is a well-known AQ derivative isolated from both terrestrial and marine sources.

The anticancer activity of physcion relies upon multiple mechanisms, including (i) the promotion of apoptosis, (ii) the perturbation of cell-cycle progression, and (iii) the suppression of metastasis and angiogenesis. Physcion also modulates cancer cell metabolism. In particular, it inhibits the 6-phosphogluconate dehydrogenase (6PGD) enzyme, usually overexpressed in cancer cells [89].

Autophagy is a strictly controlled process that allows cells to degrade damaged and/or aged proteins or organelles in order to maintain cellular homeostasis. Autophagy has a dual role in cancer: it may promote tumor cells survival, thus fostering tumor progression; in contrast, as type II programmed cell death, it can eliminate cancer cells [90]. In nasopharyngeal carcinoma (NPC) CNE2 cells, physcion induced autophagy. In this case, physcion-induced autophagy promoted apoptosis [91].

Wijesekara and colleagues isolated marine-derived physcion from the *Microsporum* sp. fungus extracted from the red alga *Lomentaria catenate* [62]. In HeLa cells, marine-derived physcion (10–100 μM) decreased cell viability in a dose-dependent manner after 24 h of treatment [62]. In the same cell line, at lower concentrations (1.25–10 μM), it activated the mitochondrial apoptotic pathway and induced the cleavage of caspase-9 and -3 [62]. Physcion-induced apoptosis in HeLa cells was associated with a dose-dependent (1.25–10 μM) increase in p53 and p21 protein expression [62]. p53 protein expression increases in response to multiple stressing stimuli such as DNA damage, where it orchestrates different cellular processes including apoptosis [92]. Most of the terrestrial AQs share the ability to induce DNA damage, including physcion [93]. Hence, apoptosis induced by the marine-derived physcion in HeLa cells could be related to its ability to damage DNA. 

In cervical cancer cells (SiHa and C33A) as well as in oral squamous carcinoma cells (HSC-3), physcion induced mixed forms of cell death: it mainly promoted necrotic cell death and to a minor extent caspase-3-independent apoptosis [93], which may be associated with its ability to induce non-canonical cell death. In this regard, physcion 8-O-β-D-glucopyranoside, the glucoside of physcion, upregulated Fe^2+^ intracellular levels and promoted lipid peroxidation, leading to ferroptosis in gastric cancer cells [94].

### 2.3. Aspergiolide A

**Figure 5:**
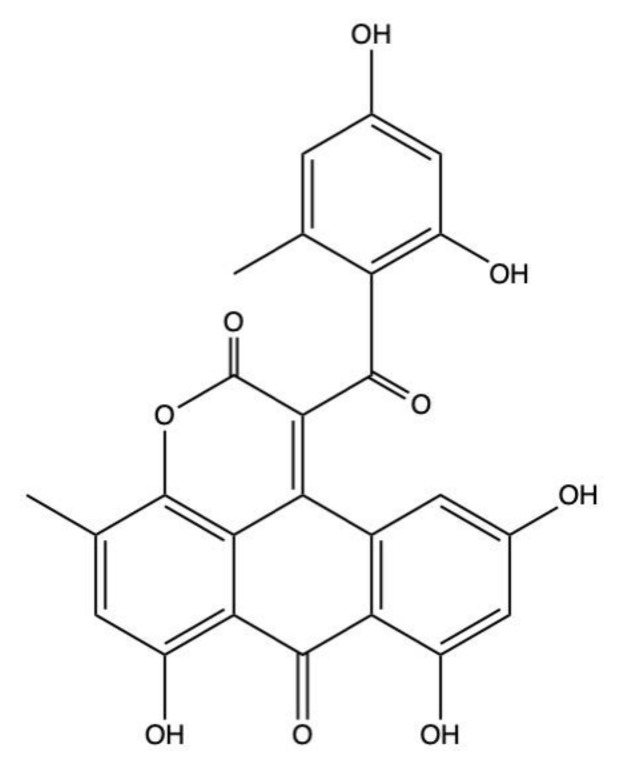
Chemical structure of aspergiolide A.

*Aspergillus glaucus* is known to produce a wide range of polyketides secondary metabolites, including the AQs 10,10’-dimer of emodin and physcion, catenarin, cynodontin, emodin, erythroglaucin, elminthosporin, physcion, questin, rubrocristin, tritisporin, and variecolorquinone A [5,27,95,96,97]. Among the secondary metabolites biosynthesized by *Aspergillus glaucus*, there is also aspergiolide A (Figure 5) and its analogues aspergiolide B–D [27,41,42,43,44].

Aspergiolide A showed a remarkable cytotoxic effect on a wide variety of solid and hematopoietic cancer cells, with an IC_50_ < 10 μM, as described in Table 1. In particular, lung adenocarcinoma A549 and HL-60 promyelocytic leukemia cells were markedly sensitive to the cytotoxic effects of aspergiolide A, displaying an IC_50_ value of 0.1 μM and 0.3 μM, respectively [41,42]. However, in mouse lymphoma P388 cells, aspergiolide A was more than 10 times less cytotoxic, with an IC_50_ value of 35 μM (Table 1). This discrepancy could be attributed to an eventual species-specific cytotoxic effect towards human cancer cells. Indeed, aspergiolide A was markedly cytotoxic not only on HL-60 cells, but even on K562 chronic myeloid leukemia cells (IC_50_: 7.5 μM) (Table 1) [41].

The anticancer mechanisms of aspergiolide A were investigated on human hepatocellular carcinoma BEL-7402 cells. Aspergiolide A (2.5–10 μM) induced caspase-dependent apoptosis, as demonstrated by the cleavage of caspase-3, -8, and -9, and PARP after 12 h treatment. Moreover, it increased the phosphorylation of histone H2AX at serine 139 (γ-H2AX), as observed from its marked-up protein expression by western blot [43]. Γ-H2AX represents the earliest cellular events triggered by DNA damage, and is, thus, commonly used as a marker of its occurrence [98]. Apart from its widely known and characterized role in DNA damage, γ-H2AX is also involved in the apoptotic process, wherein it serves as the earliest epigenetic modification during apoptosis [99]. The different pattern of γ-H2AX could be detected by immunofluorescence microscopy or FACS (fluorescence activated cell sorting) analyses, but not by western blotting [99]. For instance, confocal immunofluorescence microscopy unveiled that the staining pattern of apoptotic γ-H2AX differs from that of DNA damage response (DDR)-induced γ-H2AX. The first one is characterized by a ring staining during the early apoptotic stage and a pan-staining of the nucleus that persists until apoptotic bodies formation; the second one, instead, is characterized by a focal patterns of DNA damage foci [99]. Since Wang and colleagues analyzed γ-H2AX phosphorylation induced by aspergiolide A by western blotting, it is not possible to distinguish whether the increased γ-H2AX expression is related to apoptosis or is a response to DNA damage. However, since aspergiolide A (10–100 μM) inhibited the activity of topoisomerase II in BEL-7402 cells, the increase of γ-H2AX is most likely due to both DNA damage and the apoptosis that comes with it [43].

Aspergiolide A also displayed anticancer effects in vivo in two different hepatocellular carcinoma xenografts: Kun Ming mice inoculated with H22 mouse hepatoma cells and nude mice (strain was not indicated) inoculated with BEL-7402 human hepatoma cells [43]. The intraperitoneal administration of aspergiolide A (5 mg/kg, 15 mg/kg, or 45 mg/kg) to H22-inoculated Kun Ming mice dose-dependently inhibited the growth of subcutaneous tumors, up to 66% reduction at the highest tested dose (45 mg/kg). A similar trend was observed also in BEL-7402-inoculated nude mice intraperitoneally treated with aspergiolide A (7 mg/kg, 14 mg/kg, or 28 mg/kg) [43]. Most importantly, in both hepatocellular carcinoma xenograft models, at any tested dose, aspergiolide A did not influence the body weight of mice, differently from adriamycin (2 mg/kg), used as positive control [43].

### 2.4. Alterporriols

**Figure 6:**
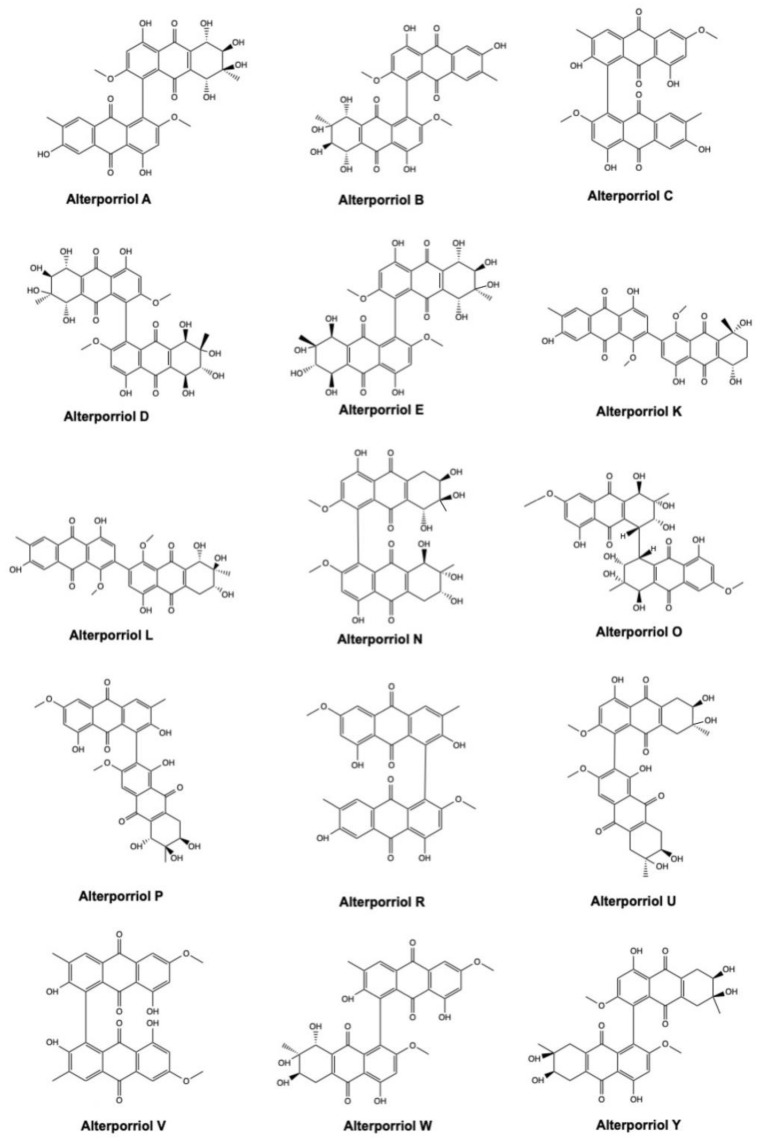
Chemical structure of alterporriol A–Y.

Alterporriols (A–Y) (Figure 6) are a group of AQ derivatives isolated from two main marine-derived fungal species: *Alternaria* and *Stemphylium* sp. (Table 1).

Despite having a similar chemical structure, alterporriols exhibited a different activity and potency in the different types of tumor models (Table 1). Noteworthy is alterporriol P, which was markedly cytotoxic on prostate PC-3 cancer cells and colon HCT-116 cancer cells, where the IC_50_ values were 6.4 μM and 8.6 μM, respectively; on hepatocellular carcinoma cells (HepG2 and Hep3B) and multidrug-resistant breast cancer cell (MCF-7/ADR), the IC_50_ were >20 μM (Table 1) [37]. These results point out that alterporriol P has tumor-specific effects, with colon and prostate cancers being the most sensitive to its cytotoxic effects.

Among all alterporriol congeners, only alterporriol L’s anticancer activity has been further characterized. Alterporriol L was cytotoxic on MCF-7 and MDA-MB-435 breast cancer cells, with IC_50_ values after 48 h treatment equal to 20.04 μM and 13.11 μM, respectively (Table 1). Moreover, it induced MCF-7 breast cancer cell death in a dose-dependent manner (10–40 μM), reaching about 64% of cell death at the highest tested concentration. Alterporriol L principally boosted necrosis rather than apoptosis. Indeed, at the concentration 20 μM, about 56% of cells were killed by alterporriol L, but about the 14% of them succumbed to apoptosis. At the same concentration (i.e., 20 μM), alterporriol L promoted apoptotic cell death by rising ROS production and intracellular Ca^2+^ levels, together with the collapse of the mitochondrial membrane potential [39]. However, the comprehensive mechanism of cell death induction of alterporriol L has not yet been elucidated.

### 2.5. Bostrycin

**Figure 7:**
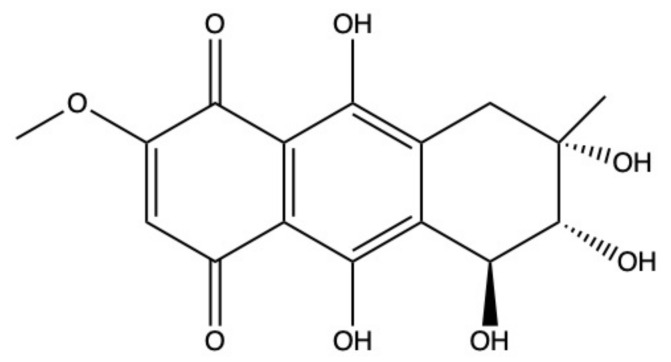
Chemical structure of bostrycin.

Bostrycin (hydroxy-methoxy-tetrahydro-5-methyl anthracenedione) (Figure 7) and two bostrycin derivatives, deoxybostrycin and 3,9’-deoxy-7-methoxybostrycin, are novel AQ derivatives isolated from different marine-derived fungal species, including the mangrove-derived *Nigrospora* sp. [50] and the sea fan-derived *Fusarium* sp. [36].

Xia and colleagues [50] found that bostrycin is cytotoxic (IC_50_ < 20 μM) on a panel of human solid tumor cell lines (Table 1) [50]. In KB (human oral epidermal carcinoma cells) and MCF-7 cells treated with bostrycin, the IC_50_ values were 4.19 μg/mL (12.46 μM) and 6.13 μg/mL (18.23 μM), respectively (Table 1) [50]. In contrast, Trisuwan and colleagues reported that the IC_50_ of bostrycin was 0.9 in KB cells and 2.7 μM on MCF-7 cells [36], hence 13.8- and 6.8-fold lower compared to the previous cited study. The main reason for this difference in terms of cytotoxicity could be a different treatment time. Neither of the studies included this information.

In A549 cells, bostrycin dose-dependently (5–20 μM) and time-dependently (24–72 h) promoted apoptosis and blocked cell-cycle progression in the G0/G1 phase. The massive accumulation of cells in the G0/G1 phase was associated with the downregulation of phospho-Akt and p110α (PI3K catalytic subunit alpha) protein expression, observed in A549 cells treated with bostrycin 10 μM for 72 h [100]. Additionally, bostrycin increased the expression of the tumor suppressor protein p27 (cyclin-dependent kinase inhibitor 1B), probably as a consequence of phospho-Akt downregulation [100].

The ability of different miRNAs to modulate the PI3K/Akt signaling pathway is widely recognized [101,102,103]. In A549 cells, bostrycin (10 μM) upregulated the expression of miRNA-638 and miRNA-923, which are involved in tumor progression [100]. Hence, it is quite possible that bostrycin downregulates the PI3K/Akt pathway through the upregulation of miRNA expression, as the authors stated. However, the correlation between these two events has to be confirmed.

### 2.6. Nidurufin

**Figure 8:**
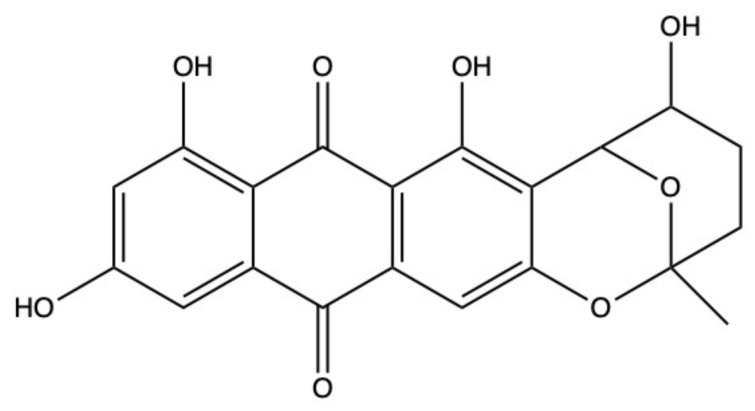
Chemical structure of nidurufin.

The AQ nidurufin (Figure 8) was isolated from the marine-derived fungi *Penicillium flavidorsum* [46] and *Aspergillus versicolor* [47,59].

In K562 cells, the anticancer activity of nidurufin was associated with its ability to perturb cell-cycle progression, as it markedly increased the percentage of cells in the G2/M phase in a dose-dependent (5–50 μM) and time-dependent (8–36 h) fashion [46]. However, the underpinned antitumor mechanisms of nidurufin have not been elucidated yet.

Norsolorinic acid is an AQ structurally related to nidurufin. It induced Fas (Fas death receptor, also known as APO/1)-mediated extrinsic apoptosis [60], as demonstrated by the increased expression of Fas and its ligand (FasL) and caspase-8 activation found on MCF-7 cells after norsolorinic acid (10 and 20 uM) treatment. Unlike nidurufin, no perturbation of cell-cycle progression was observed in MCF-7-treated cells with norsolorinic acid [60].

### 2.7. G503

**Figure 9:**
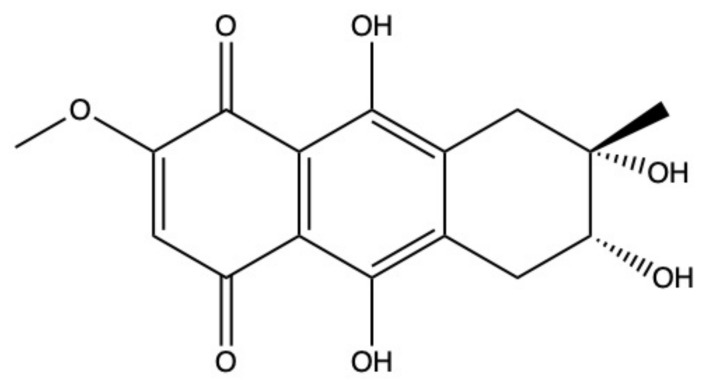
Chemical structure of G503.

G503 (Figure 9), an AQ obtained from the mangrove-derived endophytic fungus *Nigrospora* sp., exhibits cytotoxic effects on different cancer cell models with IC_50_ values ranging from 10.24 μM (SGC7901 cells) to 44 μM (retinoblastoma Rb cells) (Table 1) [58].

In SGC7901 cells, G503 dose-dependently (2.5–40 μM) and time-dependently (24–72 h) promoted apoptotic cell death. G503 (20 μM) induced the collapse of ΔΨ (mitochondrial membrane potential), probably due to the mitochondrial translocation of the pro-apoptotic protein Bax (Bcl-2-associated X protein) and the cytoplasmatic translocation of the anti-apoptotic protein Bcl-2 (B-cell lymphoma 2), observed in G503-treated cells [58]. The permeabilization of mitochondrial outer membrane is a process strictly controlled by the Bcl-2 family proteins. This protein family comprises three sub-categories: one group with anti-apoptotic functions including Bcl-2, and two groups with pro-apoptotic functions, to which belong Bax, Bak (Bcl-2 homologous antagonist/killer), and Bok (Bcl-2 related ovarian killer) proteins [104]. G503 (20 μM) also promoted the cytoplasmatic release of cytochrome *c* as well as the dose- (2.5–40 μM) and time-dependent (24–72 h) activation of caspase-9 [58]. Overall, these pieces of evidence depict the activation of the mitochondrial apoptotic pathway by G503. Moreover, G503 activated caspases-3, -8, and -9 in a dose- (2.5–40 μM) and time-dependent (24–72 h) manner in SGC7901 cells. Pre-treatment with the pan-caspase inhibitor Z-VAD-FMK and the caspase-9 inhibitor Z-LEHD-FMK reduced the apoptotic cell rate in G503-treated cells, while pre-treatment with the caspase-8 inhibitor Z-IETD-FMK did not reduce it [58]. This suggests that caspase-8, a marker of the death receptor apoptotic pathway, is not the main actor responsible for G503 pro-apoptotic activity. Moreover, G503 (20 μM) promoted the cleavage of caspase-4 [58], presuming that G503 could promote endoplasmic reticulum (ER) stress. Indeed, excessive and prolonged ER stress could promote the cleavage of caspase-4, which, therefore, prompts apoptotic cell death through the direct activation of caspase-9 without involving cytochrome *c* and/or Apaf1 (apoptosis inducing factor 1) [105]. However, activation of caspase-4 is not enough to demonstrate ER stress-induced apoptosis. Thus, currently, the pro-apoptotic activity of G503 should be mainly ascribed to its ability to perturb mitochondrial homeostasis, as long as the ability of G503 to activate ER stress and extrinsic apoptosis will be confirmed by using specific biomarkers.

G503 was tested also on non-tumoral cells, where it displayed a cytotoxic effect comparable, and in some cases even stronger, to that observed in tumor cells. The IC_50_ value in human umbilical vein endothelial (HUVEC) cells and in normal human Chang liver cells treated with G503 for 48 h was 22.4 and 17.5 μM, respectively [58], thus showing a non-selective activity towards cancer cells. This aspect should definitely be taken into account, since most of the side effects caused by anticancer drugs, including significant ones, are mainly due to their poor if not absent selectivity towards cancer cells [106].

### 2.8. SZ-685C

**Figure 10:**
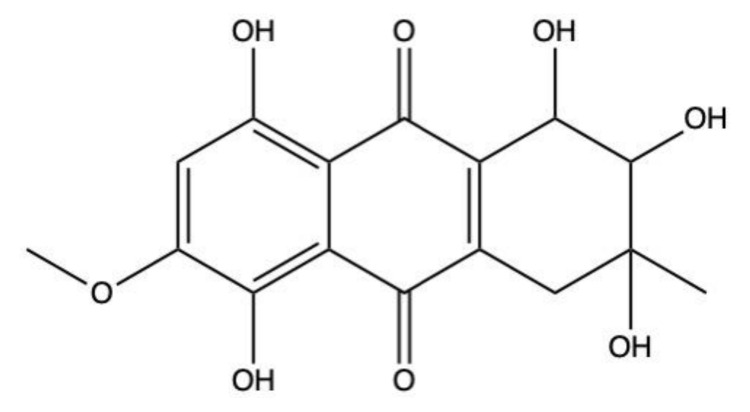
Chemical structure of SZ-685C.

The mangrove endophytic fungus *Harolosellinia* sp. is the only marine fungus producing an AQ derivative (called SZ-685) (Figure 10) identified until now.

As reported in Table 1, SZ-685C displayed a marked cytotoxic activity in multiple human and murine cancer models, with IC_50_ values < 10 μM in breast and prostate cancers, hepatocellular carcinoma, glioma, and leukemia cells (Table 1).

The anticancer potential of SZ-685 relies on two main pro-apoptotic mechanisms: the suppression of the Akt signaling pathway and the perturbation of the expression of different miRNAs involved in the control of tumor progression and apoptosis. Induction of apoptosis was recorded in all tested cancer cell models treated with SZ-685C. In radio-sensitive (CNE2) and radio-resistant (CNE2R) NPC cells, SZ-685C (10–40 μM) triggered both the intrinsic and the extrinsic apoptotic pathway [65]. In these two cancer cell models, the pro-apoptotic stimuli triggered by SZ-685C were the modulation of the Stat3 (signal transducer and activator of transcription 3)/Jabl/p27 signaling pathway and the downregulation of miRNA-205 [65]. Depending on cancer type, miRNA-205 is upregulated or downregulated, thus promoting or suppressing tumor initiation and progression [107]. In particular, the increased expression of miRNA-205 in NPC cells promotes radio-resistance by upregulating the phosphorylation of Akt through the downregulation of PTEN, the main negative regulator of Akt [107]. In both CNE2 and CNE2R cells, SZ-685C decreased the expression of miRNA-205, upregulated the protein expression of PTEN, thus downregulating the protein expression of phospho-Akt [65]. Notably, SZ-685C downregulated miRNA-205 more markedly (5-fold compared to untreated cells) in CNE2R cells. In radio-sensitive CNE2 cells, instead, the downregulation of miRNA-205 was about 2-fold compared to untreated cells, probably because the basal expression of miRNA-205 in the latter was lower compared to the radio-resistant counterpart [65].

As mentioned before, miRNAs are tumor specific. In pituitary adenoma, several miRNAs are aberrantly expressed either downregulated or upregulated. Among all, miRNA-200c acts as an oncogene and inhibits apoptosis through the modulation of the PTEN/Akt pathway [108]. SZ-6585C promoted apoptosis in pituitary adenoma cells through the suppression of the PTEN/Akt signaling pathway. This effect was partially due to miRNA-200c downregulation. Indeed, the dose-dependent downregulation of miRNA-200c by SZ-685C (7.5–30 μM) was responsible for its pro-apoptotic activity in rat MMQ pituitary adenoma cells [64]. Moreover, a later study demonstrated that SZ-685C dose-dependently (5–20 μM) decreased the phosphorylation of Akt through the upregulation of PTEN expression and induced apoptosis in primary human NFPA (nonfunctioning pituitary adenoma) cells [63].

In both adriamycin-sensitive estrogen receptor-positive MCF-7 cells and adriamycin-resistant MCF-7/ADR cells, as well as in estrogen receptor-negative MDA-MB-435 cells, SZ-685C (1.5–15 μM) activated the intrinsic and the extrinsic apoptotic pathway [66]. As mentioned before, a pivotal role in SZ-685C-induced apoptosis was the suppression of the Akt signaling. Since Akt could suppress apoptosis by phosphorylating different downstream mediators, such as Bad, Bcl-xL (B-cell lymphoma-extralarge), FoxOs, and pro-caspase-9, suppression of Akt signaling could promote apoptosis [109]. In MCF-7/ADR and MCF-7/Akt (MCF-7 cells that constitutively express active Akt), the downregulation of phospho-Akt by SZ-685C led to a dose-dependent (2–8 μM) decrease in phospho-Bad and Bcl-xL protein expression, which promoted cancer cell apoptosis [66]. Intraperitoneal administration of SZ685-C (50 mg/kg, every 4 days for 30 days) also decreased the expression of phospho-Akt and Bcl-xL in vivo, which resulted in the inhibition of the growth of MCF-7/ADR xenografted tumors in female BALB/c-nu mice [66].

FoxO1 and FoxO3a are two downstream mediators of the Akt pathway. After being activated through phosphorylation, they inhibit the activity of some apoptotic proteins, such as Bim (Bcl-2-interacting mediator of cell death), and promote cell survival [110]. In MCF-7 and MDA-MB-435 breast cancer cells, SZ-685C-induced apoptosis was associated with the suppression of Akt phosphorylation, which in turn induced a decrease in phospho-FoxO1 and phospho-FoxO3a protein expression, finally resulting in increased Bim protein expression [67].

Overall, all these findings point out that SZ-685C could be an effective anticancer agent, being active also on different resistant cancer models, as observed in both in vitro and in vivo studies. 

In vitro, SZ-685C dose-dependently and time-dependently decreased radio-resistant NPC cell viability almost to the same extent as in the radio-sensitive counterpart [65]. Moreover, SZ-685C was quite effective on various adriamycin-resistant cancer cell lines. Indeed, the AQ metabolite decreased the adriamycin resistance factor (i.e., the ratio of IC_50_ of adriamycin in resistant cells to the adriamycin IC_50_ in sensitive cells) from 19.19 to 0.57 in MCF-7 cells, from 58.33 to 1.24 in K562 cells, and from 54.94 to 0.91 in HL-60 cells [66]. In vivo, intraperitoneal administration of SZ-685C (50 mg/kg, every 3 days for 35 days) significantly inhibited the tumor growth of MDA-MB-435 xenografted female BALB/c-nu mice, without inducing toxic effects as loss of body weight [67]. The same inhibitory effect was observed on MCF-7/ADR xenografted BALB/c-nu mice intraperitoneally administered with SZ-685C (50 mg/kg, every 4 days for 30 days), hence showing that it could also override adriamycin resistance in vivo. Again, no detectable toxic effects were found in SZ-685C-treated mice, in contrast to a significant loss of body weight observed in adriamycin-treated mice [66]. Although preliminary, this finding leads to suppose that SZ-685 could have a safer toxicological profile compared to adryamycin. Accordingly, in vitro data already pointed out a partial selectivity of action of SZ-685C towards tumor cells. In rat normal pituitary cells, the IC_50_ value was about 3.8- and 3-fold higher compared to that calculated in rat adenoma MMQ cells (14.51 μM at 24 h and 13.2 μM at 48 h) and in human primary adenoma cells (18.76 μM at 24 h) (Table 1) [63,64].

### 2.9. 1403P-3

**Figure 11:**
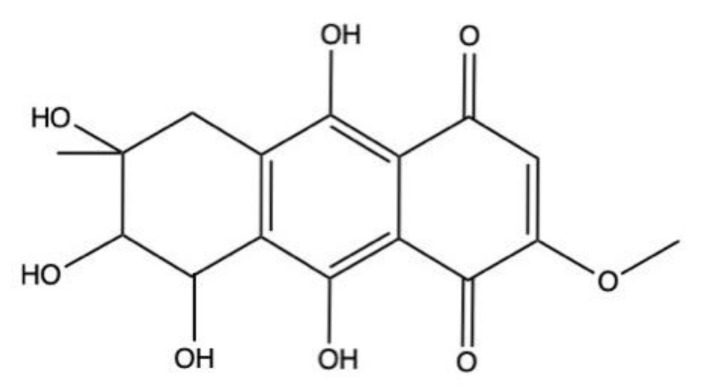
Chemical structure of 1403P-3.

The anthracenedione derivative 1403P-3 (Figure 11), isolated from the mangrove endophytic fungus no. 1403, exhibited almost an equal cytotoxic effect on both KB and KBv200 (multidrug resistant) cells, with IC_50_ values after 72 h treatment of 19.66 μM and 19.27 μM, respectively (Table 1) [31].

On both KB and KBv200 cells, 1403-P dose-dependently (18–144 μM) and time-dependently (12–48 h) induced intrinsic and extrinsic apoptosis. In particular, 1403P-3 promoted the caspase-8-dependent cleavage of caspase-2 and Bid, a protein belonging to the so-called “BH3 domain only” pro-apoptotic protein family, in a time- and dose-dependent fashion [31]. This latter event is considered critical in linking the two apoptotic pathways [111]. Indeed, when Bid is cleaved into tBid upon caspase-8 activation, it translocates to the mitochondria membrane, where it inactivates Bcl-2 protein and activates Bax protein, hence promoting cell death pathways [111].

Very similar effects were recorded on MCF-7 breast cancer cells, where 1403P-3 activated both the intrinsic and extrinsic apoptotic pathways [32]. In this latter experimental model, 1403P-3 blocked Akt phosphorylation in a time- (12–48 h) and dose-dependent (4.75–19 μM) manner [32].

## 3. Anticancer Mechanisms of AQs from Other Marine Sources

Besides marine fungi, AQs were isolated from other marine microorganisms and organisms. As shown in Table 2, the main source of marine AQs is the bacterial species *Streptomyces*, one of the largest genera of Actinobacteria, isolated from marine sediment, marine plants, and sponges. Additionally, some AQs have been isolated directly from sponges and crynoids. For instance, the AQs derivatives (S)-(−)-rhodoptilometrin and 1’-dehydroxyrhodoptilometrin were isolated from the echinoderms *Colobometra perspinosa* and *Comanthus* sp. [112,113], while rhodocomatulin 5,7-dimethylether was isolated from the sponge *Clathria hirsuta* and the crinoid *Comatula* (*Validia*) *rotalaria* Lamarck [114].

As reported in Table 2, most of the AQs isolated from non-fungal sources exhibited cytotoxic and/or antiproliferative effects towards cancer cells only at concentrations greater than 50 μM, while very few AQs displayed these anticancer effects in the range of nanomolar concentrations (Table 2). Herein, we describe the antitumor mechanisms of the few characterized AQs from non-fungal marine sources.

### 3.1. 1’-Deoxyrhodoptilometrin and (S)-(−)-Rhodoptilometrin

**Figure 12:**
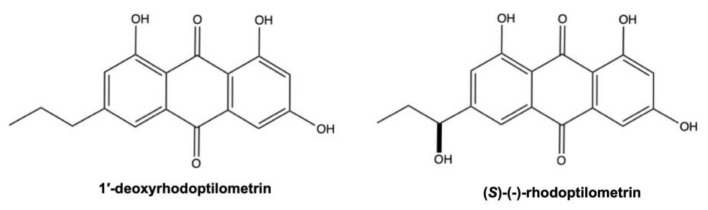
Chemical structure of 1’-deoxyrhodoptilometrin and (*S*)-(−)-rhodoptilometrin.

Although 1’-deoxyrhodoptilometrin and (*S*)-(−)-rhodoptilometrin are structurally similar (Figure 12), they induce different cell death mechanisms, which also differ depending on tumor type. On C6 rat glioma cells, 1’-deoxyrhodoptilometrin (25 and 50 μM, for 24 h) promoted apoptotic and necrotic cell death; (*S*)-(−)-rhodoptilometrin, instead, did not trigger apoptosis at the same doses and treatment time, while at the highest-tested dose (50 μM), it slightly induced necrotic cell death. In contrast, on HCT-116 cells 1’-deoxyrhodoptilometrin and (*S*)-(−)-rhodoptilometrin did not induce neither apoptosis nor necrosis after 24 h treatment [113]. Wätjen and colleagues stated that the presence of hydroxyl group at C-1’ position of (*S*)-(−)-rhodoptilometrin is responsible for its lower cytotoxic effect compared to 1’-deoxyrhodoptilometrin [113].

The antitumor mechanism of these two AQs does not involve ROS generation, but instead a potent inhibitory activity against different protein kinases playing a role in cell proliferation, metastasis, and angiogenesis. 1’-deoxyrhodoptilometrin and (*S*)-(−)-rhodoptilometrin (25 and 50 μM) markedly suppressed ERK (extracellular signal-regulated kinase) phosphorylation in C6 cells [113]. Moreover, the two AQs derivatives strongly inhibited the activity of multiple kinases, as observed by using an acellular protein kinase activity assay. In particular, 1’-deoxyrhodoptilometrin and (*S*)-(−)-rhodoptilometrin strongly suppressed the activity of protein kinases Aurora A and B, which are cyclin-dependent kinases involved in cell-cycle regulation. Additionally, by using the same acellular assay, the authors found that both compounds, but in particular 1’-deoxyrhodoptilometrin, also inhibited the activity of protein kinases involved in metastasis and angiogenesis, including the focal adhesion kinase (FAK) (KIC_50_ (i.e., the concentration producing 50% protein kinase activity inhibition): 8.4 μM), the vascular endothelial growth factor (VEGF) receptor 2 kinase (KIC_50_: 1.8 μM), and the epidermal growth factor (EGF) receptor kinase (KIC_50_: 4 μM) [113]. 1’-deoxyrhodoptilometrin and (*S*)-(−)-rhodoptilometrin are structurally related to emodin: if emodin possesses a methyl moiety on position 2, 1’-deoxyrhodoptilometrin and (*S*)-(−)-rhodoptilometrin have, instead, a propyl and a 1-hydroxypropyl moiety, respectively [113]. Since emodin is considered a potent natural protein kinase inhibitor, the ability of these two marine AQs to inhibit several protein kinases is not surprising. Indeed, emodin suppressed the activity of multiple kinases as PKC (protein kinase C), CK2 (casein kinase I), and the tyrosine kinase Her-2/neu, MAPK, and ERK [129,130]. Additionally, emodin showed anti-invasive effect by inhibiting FAK and VEGF, as 1’-deoxyrhodoptilometrin and (*S*)-(−)-rhodoptilometrin.

### 3.2. Tetracenomycin D and Heliomycin

**Figure 13:**
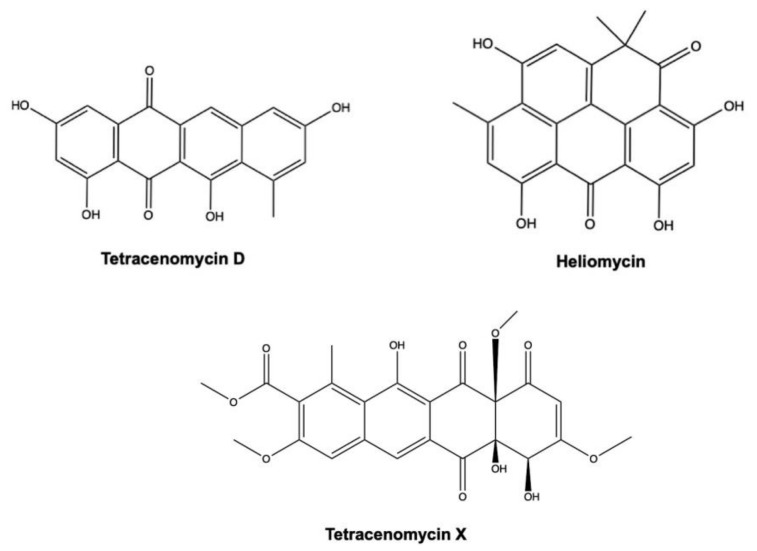
Chemical structure of tetracenomycin D, heliomycin and tetracenomycin X.

Tetracenomycin D (or tetracynomycin D) and heliomycin (also known as resistomycin) (Figure 13) are two newly discovered marine AQs extracted from *Streptomyces* spp. isolated from marine sediment [126] and marine sponge *Pseudoceratina arabica* [131].

Both AQ derivatives exhibited a strong cytotoxic effect on HMO2 gastric cancer cells and HepG2 cells, with IC_50_ values equal or lower than 0.013 μg/mL (Table 2) [126].

A recent study investigated the antitumor activity of tetracenomycin X in two different experimental models of lung cancer: H460 xenografts in BALB/c nude mice and A549 and H460 cell lines. In the in vivo model, tetracenomycin X decreased the volume of H460 xenografts in nude mice (antitumor rate: 42%); in the A549 and H460 cells, it induced a cell-cycle arrest at the G0/G1 phase mediated by a decrease in cyclin D1 and CDK4. Of note, tetracenomycin X reduced cyclin D1 levels through a dual mechanism: induction of its proteasomal degradation and activation of p38 and c-JUN [132].

Tetracenomycin D and heliomycin act as epigenetic modulators. Epigenetic modifications include DNA methylation, histone acetylation, and small RNA-mediated gene silencing. All of them could affect the expression of several, if not all, genes implicated in cancer initiation and progression [133]. Histone deacetylases (HDACs) are a class of enzymes which control histone acetylation by removing an acetyl moiety of lysine in histone N-terminal regions [133]. Besides their deacetylating histones activity, HDACs have other non-histone-related biological activities including the modulation of (i) cell-cycle progression, (ii) autophagy, (iii) DNA damage processes, and finally (iv) apoptosis [134]. Saleh Abdelfattah and colleagues explored the histone deacetylase inhibitory activity of tetracenomycin D and heliomycin using a HDAC colorimetric assay performed on the nuclear extract of HeLa cells. The concentrations producing 50% inhibition of HDAC activity were 10.9 μg/mL for tetracenomycin D and 29.8 μg/mL for heliomycin [131]. Using a computational docking study, the same authors found that the two compounds have a good binding interaction with HDAC2 and HDAC3 [131].

### 3.3. Galvaquinones

**Figure 14:**
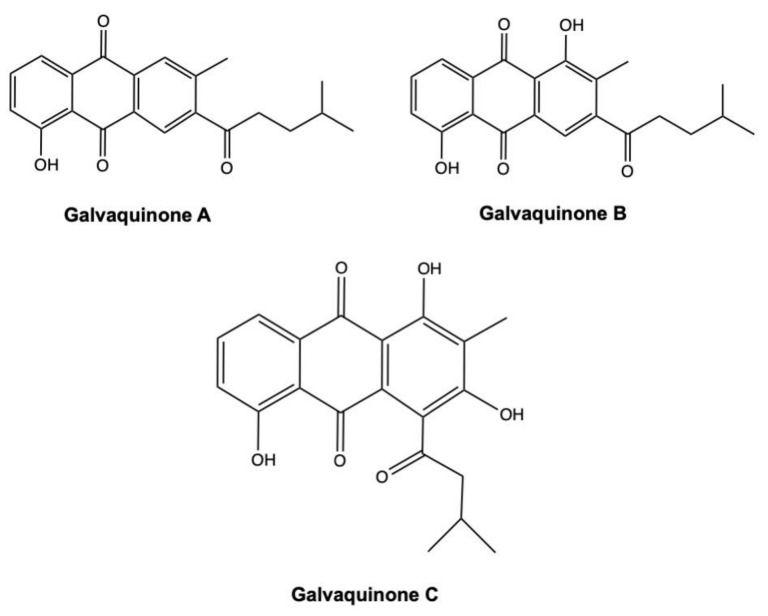
Chemical structure of galvaquinone A–C.

Galvaquinones A–C (Figure 14) are three new AQs derivates isolated from the marine sediment-derived *Streptomyces spinoverrucosus* [121].

The IC_50_ values on Calu-3 and H2287 non-small cell lung cancer (NSCLC) cells treated with galvaquinone A and C for 96 h were >50 μM (Table 2). On the same cancer cell models, in contrast, galvaquinone B displayed IC_50_ values of 12.2 and 5 μM, respectively (Table 2) [121]. The stronger cytotoxicity of galvaquinone B reflects a more prominent epigenetic modulatory activity. Indeed, in the Locus DeRepression assay, galvaquinone B strongly inhibited HDAC activity at both 1 μM and 10 μM concentrations, while galvaquinone A inhibited HDAC activity only at 10 μM, and also to a far less extent [121]. To date, several natural HDAC inhibitors are highly effective in suppressing tumor cells proliferation [135]. These include largazole, isolated from the marine cyanobacterium *Symploca* sp. [136], and chromopeptide A, extracted from the marine sediment-derived *Chromobacterium* sp. [137].

### 3.4. Angucyclines

Angucyclines are a class of aromatic polyketides composed by an angular benz[a]anthracene scaffold (aglycone) mostly linked to C-glycosidic moiety. Angucyclines and their aglucones (angucyclinones) are produced exclusively by terrestrial and marine actinomycetes, in particular those of the *Streptomyces* species. Notably, most of angucyclines are found in nature as glycosides [138,139].

**Figure 15:**
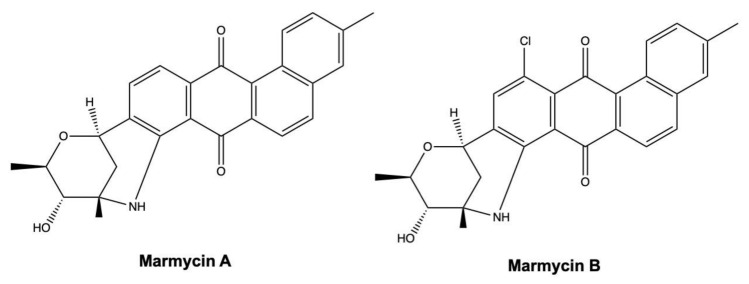
Chemical structure of marmycin A and marmycin B.

As described in Table 2, different marine-derived angucyclines, such as marmycin A, display a marked cytotoxic effect on cancer cells with IC_50_ values in the nanomolar range (Table 2). Marmycin A, together with its halogenated analogue marmycin B (Figure 15), differs from most of angucycline congeners. Indeed, they display both C- and N-glycoside bonds, whereas most of the other anglucyclines present a C-glycoside link [125].

Marmycin A and B were highly cytotoxic on HCT-116 colon cancer cells, with an IC_50_ value after 72 h of 0.06 μM for marmycin A, being almost 18-fold more cytotoxic than its halogenated congener marmycin B (IC_50_: 1.09 μM) (Table 2) [125]. In addition, in ovarian A2780 cancer cells, marmycin A (20–200 nM) consistently blocked cell cycle in the phase G0/G1 and promoted apoptosis [125].

**Figure 16:**
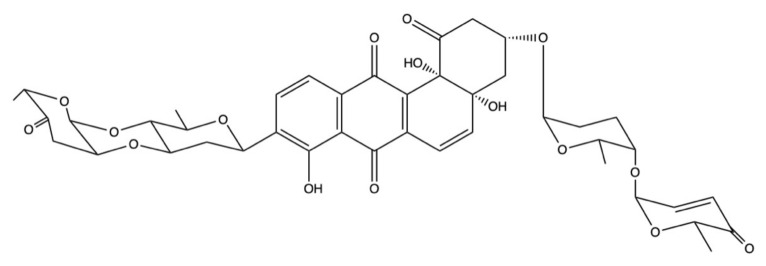
Chemical structure of saquayamycin B.

Saquayamycin B (Figure 16) is another angucycline glycoside, isolated from two different *Streptomyces* strains derived from deep sea sediment [119,120,123].

On three different breast cancer cell lines (MCF-7, MDA-MB-231, and BT-474), saquayamycin B displayed a marked cytotoxic effect with IC_50_ equal to 0.40, 0.38, and 0.41 μM, respectively (Table 2). Besides suppressing cell proliferation, saquayamycin B (0.025 and 0.050 μM) also inhibited the invasion and migration of MDA-MB-231 cells after 12 h of treatment [119]. A slightly higher cytotoxicity was observed in HepG2 and plc-prf-5 hepatocellular carcinoma cells, where saquayamycin B displayed an IC_50_ of 0.14 and 0.24 μM (Table 2). On human T-lymphoblastic Jurkat cells, but also on SMMC-7721 hepatoma cells, instead, the IC_50_ of saquayamycin B was almost one order of magnitude lower than those obtained on the hepatic and breast cancer cell lines (Table 2) [120,123]. Notably, on SMMC-7721 cells, saquayamycin B (0.025–0.100 μM) dose-dependently promoted apoptosis [120]. However, as for marmycin A, the authors did not investigate the underlying pro-apoptotic mechanisms of saquayamycin B.

## 4. Genotoxicity of AQs

Genotoxicity is one of the most critical factors to be considered for AQs. Genotoxicity is the ability of chemicals to interact with nucleic acids and/or cause DNA or chromosomal damage, with these events occurring at dose levels where the substance is not cytotoxic. The genetic alterations can involve somatic or germ cells, in the latter case inducing heritable changes [140]. Genotoxicity plays a critical role in cancer development, and in a variety of other disorders, such as neurodegenerative diseases, cardiomyopathies, or atherosclerosis [141,142].

Notwithstanding the huge number of studies on AQs’ genotoxicity and carcinogenicity, data are not conclusive. Independently from AQs’ origin, terrestrial or marine-derived, caution is necessary to definitively assess their risk/benefit profile before conceiving their clinical use as anticancer agents.

AQs have been routinely used for decades as natural dyes or drugs [5]; for more than 30 years, many studies have attempted to determine their genotoxic potential, which is currently debated [5,143]. Recently, the EFSA (European Food and Safety Authority) provided a scientific opinion on the possible harmful effects on health of long-term use of AQs present in dietary products, generally used as laxatives [144]. The main concern was the possible association of AQs consumption with colon cancer. However, a safe daily intake for AQ products was not defined for the general population. The EFSA report states that “AQs should be considered genotoxic and carcinogenic unless there are sufficient evidence on the contrary” [144]. A recent meta-analysis tried to quantify the risk of colon cancer associated with AQ consumption considering both active compounds (emodin, aloe-emodin, physhion, chrysophanol, rhein, danthron, senna glycosides) and herbal laxative preparations. Interestingly, the study highlighted how the risk is greater for self-administered products, available without medical prescription [145]. One reason may be the mistaken belief that “natural means safe,” which prompts the use of these products above the recommended dose, underestimating the risk for health. On the contrary, “natural” laxatives bring many risks and, not without reason, their use for more than 2 weeks requires medical supervision [144].

Emodin is one of the most characterized compounds of the AQ family. Analyzing the vast literature, it is not clear whether emodin is genotoxic and a carcinogen. Indeed, many genotoxicity and mutagenicity studies have been carried out in the past 25 years, showing contrasting results. Another aspect that further complicates the state of the play is, as highlighted above, emodin’s antimutagenic activity [88]. This means that, under differing conditions of cell type or dose, emodin can act as a mutagen or an antimutagen. The ability of natural products to display both mutagenic and anti-mutagenic nature is not a novelty: β-carotene is a consolidated example of these compounds, also called “Janus mutagens”, from the Roman god with one head and two faces looking in opposite directions [84].

Emodin is characterized by a planar AQ core, which can be embedded in the DNA double helix [146,147]. One of the first studies on emodin genotoxicity was carried out in 1996 by Muller and colleagues, disclosing its ability to induce DNA damage [147]. The mechanism was clearly unraveled and includes both direct and ROS-mediated, hence indirect, DNA interactions [93,147]. Emodin acted as a clastogen, inducing structural chromosomal aberration, and presumably as a DNA intercalator. In common with DNA intercalating agents such as anthracyclines, emodin inhibited topoisomerase II, as demonstrated using a decatenation assay [147,148], and catalytically inhibited the cleavable complex, poisoning topoisomerase II. Aloe-emodin and danthron share the same mechanism [147]. Emodin showed the most potent inhibition on the isolated enzyme, while it showed the weakest activity in cell culture assay compared to danthron and aloe-emodin [147]. The possible explanations for those different effects are (1) a different interaction with cell medium or cellular components among the three molecules before reaching the target or (2) a different specificity for the two topoisomerase II subtypes α and β [147]. Only one of the two subtypes (α) is used in the decatenation assay and emodin could have higher affinity for this subtype [147].

Several others in vitro studies have been carried out on emodin, pointing to a genotoxic activity. For instance, induction of gene mutations and micronuclei were investigated after treatment with emodin for 4 h at concentrations up to 111 µM in the mouse lymphoma L5178Y cells, in absence of S9 metabolic activation. Gene mutation experiments were performed in the thymidine kinase (TK)+/− locus of L5178Y cells, which is considered one of the most sensitive loci to detect any mutations [149]. An increase in gene mutation frequency and micronuclei formation were observed compared to negative control [147]. A later study by Chen and colleagues [150] showed that 40 µM emodin induced DNA damage (tested by comet assay) after long-term treatment (24 h) in human tongue cancer (SCC-4) cells and inhibited mRNA expression of genes associated with DNA damage and repair, such as the checkpoint kinases ATM (ataxia telangiectasia mutated) and ATR (ataxia-telangiectasia and Rad3-related), which are central regulators of DNA damage response. Moreover, emodin downregulated the tumor suppressor BRCA1 (breast cancer associated gene 1), which is usually activated by DNA double strand breaks, and MGMT (O6-methylguanine DNA methyltransferase), which protects from damages of alkylating agents [150]. However, the results are not convincing. The reasons for concern are the lack of data at a shorter treatment time, as the *OECD* (Organization for Economic Co-operation and Development) Genetic Toxicology Test *Guidelines* recommend [151]. Indeed, the elevated cytotoxicity measured after 24 h could be linked to excessive DNA damage and may have interfered with comet analysis, giving a false positive result.

Emodin genotoxic potential has been observed also after cytochrome P450-dependent biotransformation. Exposure of emodin to rat liver microsomes’ fraction generated 2-hydroxyemodin and ω-hydroxyemodin. 2-Hydroxyemodin induced much higher micronucleus frequencies compared to the parent dihydroxyantraquinone emodin [152]. In contrast, ω-hydroxyemodin, derived from the hydroxylation of the exocyclic methyl group of emodin, showed lower potential for the induction of micronuclei compared to emodin [152].

However, as anticipated above, besides the evidence about emodin’s genotoxicity, there are also reports showing contrasting results [153,154]. For instance, in mammalian test systems using V79 Chinese hamster cells, no genotoxicity was found either with or without metabolic activation up to 111 µM emodin [155]. Moreover, emodin did not increase the micronuclei frequency in human lymphocytes with and without metabolic activation nor in the metabolically competent human hepatoma cell line HepG2 [156].

The frequency of micronuclei was analyzed after single oral gavage administration of emodin 2000 mg/kg bw in NMRI male and female mice. Animals were sacrificed at 1 and 3 h from treatment and bone marrow cells were collected and analyzed for micronuclei induction. No increase in micronuclei frequency was recorded [157]. Out of the recommendations of *OECD* Genetic Toxicology Test *Guidelines*, only one emodin dose was tested. Moreover, the same dose did not show evidence of toxicity on bone marrow cells, suggesting an insufficient exposure of target tissue and, thus, casting doubts on the obtained negative results. The inefficient exposure could be explained by emodin pharmacokinetics, which is characterized by poor intestinal absorption, fast elimination, and low in vivo bioavailability [158].

The structure similarity of emodin with the well-known genotoxin danthron (1,8-dihydroxyanthraquinone) [18] fostered prospective evaluation for emodin carcinogenicity through the read across approach Computer-Optimized Molecular Parametric Analysis of Chemical Toxicity (COMPACT) procedure of the National Toxicology Program (NTP) of the National Cancer Institute (NCI). Emodin was predicted positive for carcinogenicity [159].

The contrasting results between the in vitro tests and the computational prediction prompted a clarification of the potential carcinogenic properties of emodin by in vivo investigation. Two-year carcinogenesis studies were conducted by the NTP. Male F344/N rats were exposed for 105 weeks to average daily emodin doses of approximately 110, 320, or 1000 mg/kg bw; female rats to 120, 370, or 1100 mg/kg bw. Moreover, male B6C3F mice were exposed to average daily doses of approximately 15, 35, or 70 mg/kg bw; female mice to 30, 60, or 120 mg/kg bw. The results showed no evidence of carcinogenic activity for emodin in male F344/N rats and female B6C3F mice. In contrast, equivocal evidence of carcinogenic activity was recorded in female 344/N rats, based on a marginal increase in the incidence of Zymbal’s gland carcinoma, and in male B6C3F mice, based on a low incidence of uncommon renal tubule neoplasms [160]. Interestingly, no association with gastrointestinal cancers was recorded neither in rats nor in mice after emodin exposure at the tested daily dietary intake. This could be explained by the differences in pharmacokinetics and biological effects between humans and rodents. For instance, emodin is not found as a cathartic in rodents, possibly due to a different gut structure from humans and with certainly different reabsorption capability in colonic function.

Overall, there is no consensus regarding the mutagenicity for emodin [18]. The experimental design of some studies carried out on emodin does not comply with OECD Genetic Toxicology Guidelines (i.e., time of exposure, dosing). On the whole, the mutagenicity of emodin, whether confirmed, may depend not only on emodin per se but also on the activity of its metabolites.

Fewer but clearer results are available for the genotoxicity of danthron, also known as chrysazin. The FDA initially developed and approved its use as drug for constipation, but it was withdrawn in 1999 due to its risk of carcinogenesis. In 1990, the IARC (International Agency For Research On Cancer) classified danthron in category 2B (probably carcinogenic to humans) with the colon as the target organ both in mice and rats [161]. Danthron genotoxicity is mediated by direct DNA damage, such as topoisomerase II poisoning, DNA double-strand breaks, gene mutations, and DNA intercalation. Furthermore, danthron is responsible for dose-dependent ROS generation, 8-hydroxydeoxyguanosine formation, and depletion of glutathione [147,162,163,164,165,166,167]. Thus, danthron can also induce a ROS-mediated DNA damage.

Zhang and colleagues demonstrated that danthron can be metabolically activated. Its metabolism involves pathways other than cytochrome P450, such as the quinones dehydrogenase DT-diaphorase. Indeed, danthron (25–100 µg/mL) increased micronuclei frequency and DNA damage in Balb/c 3T3 cells, and gene mutations in *Salmonella typhimurium* TA102 strain [162]. However, the addition of dicoumarol, a DT-diaphorase inhibitor, reduced gene mutations by 27–39% [162].

In vivo studies [168,169] investigated danthron’s carcinogenicity in the cecum and colon of male rats and mice: ACI (an inbred line derived from August and Copenhagen strains) rats were fed with a basal diet containing 1% danthron and C3H/HeN mice with 0.2% danthron for 540 days. According to EFSA guidelines [170], these dietary concentrations were equivalent to 520 mg/danthron/kg bw per day for rats and 300 mg/danthron/kg bw per day for mice. Rats treated with danthron developed mucosal hyperplasia in the colon and caecum that were histologically classified as adenomas or adenocarcinomas. Moreover, pre-neoplastic lesions of the glandular epithelium of colon and caecum were detected in rats, whereas any pathological change was observed in the control group in any organ. Although pathological changes were observed in the liver of treated rats, no liver tumors were recorded [168]. In contrast, danthron induced hyperplastic lesions in the large intestine and increased number of liver adenomas and carcinomas in treated mice compared to controls [169]. These data indicate that danthron was carcinogenic under the tested conditions on the gastrointestinal tract of rats and mice and enhanced the progression of spontaneously occurring cancer in the liver of mice.

The natural occurring dimeric AQs chrysophanol and physcion are two compounds frequently isolated from fungus culture and attract attention for their pharmacological effects, including anticancer potential [91,171]. In common with previously described AQs, physcion induced DNA damage through increasing intracellular oxidative stress [93], while chrysophanol intercalated into the DNA double helix. Chrysophanol and physcion showed no significant genotoxic effects in comet assay and mouse lymphoma L5178Y locus TK+/− [167]. Nevertheless, chrysophanol exhibited strong mutagenicity in two *Salmonella* strains, TA2637 and TA1537, with and without metabolic activation [172,173,174]. The capability of chrysophanol to induce chromosomal aberration was investigated in the Chinese hamster ovary cell assay, with and without S9 metabolic activation, up to its limit of solubility (30 mg/mL). No significant increase was observed in the percentage of chromosomal aberrations [175]. Although no clastogenic activity was recorded for chrysophanol [175], a genotoxification pathway relevant for in vivo exposure has been demonstrated. Chrysophanol is metabolized by cytochrome P450-dependent reaction in the genotoxin aloe-emodin [147,152], which induced significantly higher micronuclei than chrysophanol in mouse lymphoma L5178Y cells [152]. A previous study showed that aloe-emodin shares with emodin and danthron clastogenic activity and, as mentioned before, the ability to poison topoisomerase II enzyme [147]. Several other in vitro studies showed positive results on the genotoxicity of aloe-emodin itself through different tests and in the presence or absence of metabolic activation [147,176,177] and, most interestingly, in vivo data are available for aloe-emodin. A first study assessed induction of micronuclei and structural chromosomal aberrations in Wistar male and female rats [176]. No micronuclei were recorded after oral gavage of a single dose of 1500 mg/kg bw. Negative output was also recorded for chromosome aberration after oral gavage administration of 200, 666, or 2000 mg/kg bw aloe-emodin. However, some critical issues question the reliability of the aforementioned negative results [144]. Indeed, there are no evidence that the target tissues, meaning bone marrow cells, were sufficiently exposed to the potential genotoxin, because no toxicity was observed on these cells. Moreover, an insufficient number of cells (1000 polychromatic erythrocytes (PCE)/animal against 4000 currently requested from the guidelines) was scored and only one dose was tested for the evaluation of micronuclei induction. In compliance with the OECD guidelines, Nesslany and colleagues disclosed the capability of aloe-emodin to induce DNA fragmentation in male OF1 mice [178]. In particular, mice were treated with 500, 1000, or 2000 mg/kg bw of aloe-emodin by oral gavage as suspension in 0.5% carboxymethylcellulose. A dose-dependent DNA damage was recorded in both kidney and colon cells analyzed between 3 and 6 h after treatment. Of note, positive controls, meaning either streptozotocin at 20 mg/kg intravenously (positive control for kidney cells) or orally dimethylhydrazine at 20 mg/kg bw (positive control for colon cells), were included in the study, giving a high weight to these results. Thus, the evidence on the genotoxicity of aloe-emodin was obtained in colon and kidney cells, which are consistent with the carcinogenic effects in kidney and gastrointestinal tract results of the structurally related emodin and danthron [160,169]. The pharmacokinetic profile of aloe-emodin indicates that it has a high binding activity to different organs, including kidney and liver, where it is quickly metabolized to aloe-emodin glucuronides and rhein sulfates or glucuronides [179] and then excreted in bile or urine [180]. The lipophilic AQ rhein, differently from aloe-emodin, is devoid of any genotoxic activity. Its non-genotoxic nature was established through a battery of in vitro and in vivo assays [176,181,182].

Among AQs of marine origin, aspergiolide A shares with the aforementioned compounds the ability to inhibit topoisomerase II. Its topoisomerase inhibitory activity was comparable to that of adryamicin, an antitumor agent currently used in the clinic. Micronuclei induction by aspergiolide A was assessed. The AQ was intraperitoneally administered to Kun Ming mice at 100 or 400 mg/kg bw for 30 h. Under those tested conditions, aspergiolide A did not cause any significant increase in micronuclei compared to vehicle control, suggesting a lack of genotoxic potential [43].

Taken together, these data suggest that AQs may or may not induce DNA damage and if so, they act through different mechanisms. Biotransformation represents a critical point that needs to be taken into consideration in the definition of AQs genotoxic and carcinogenic profile since the genotoxic potency is quite different among metabolites. In vivo experiments can offer a more reliable picture in the assessment of their genotoxic potential. Moreover, a dose–response analysis and a clear definition of the mechanisms of the genetic toxicity of each compound, including new compounds from marine sources, need to be fully elucidated to definitely assess their full pharmacological potential.

## 5. Conclusions

Natural compounds from marine sources are nowadays of great interest for their distinct scaffold and their diversified bioactivities. The class of AQs is characterized by large structural diversity, pronounced biological activity, and apparently low toxicity [5,8]. The structural similarity of AQ aglycons to the well-established anticancer drugs anthracyclines allowed the development from laxative, colorants, or food additives to anticancer agents.

**Figure 17:**
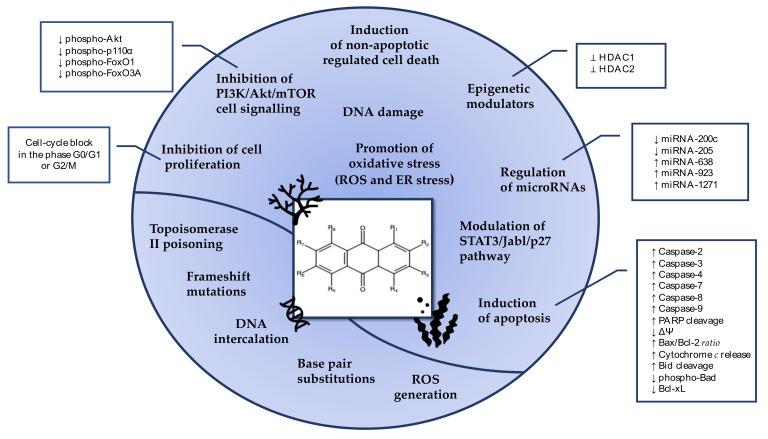
Cellular and molecular mechanisms modulated by AQs. ⊥: inhibition; ↓: decrease; ↑: increase; Akt: protein kinase B; Bax: Bcl-2-associated X protein: Bcl-2: B-cell lymphoma 2; Bcl-xL: B-cell lymphoma-extra large; ER: endoplasmic reticulum; FoxO1: forkhead box O1; FoxO3A: F forkhead box O3A; HDAC: histone deacetylase; ROS: reactive oxygen species; STAT3: signal transducer and activator of transcription 3; p27: cyclin-dependent kinase inhibitor 1B; PARP: poly-(ADP-ribose)-polymerase; PI3K: phosphoinositide 3-kinase; mTOR: mammalian target of rapamycin; ΔΨ: mitochondrial membrane potential.

As described in Figure 17, the anticancer activity of marine-derived AQs mainly relies on their ability to induce DNA damage, cell-cycle arrest, and apoptosis [17]. The cytotoxic and cytostatic activity of many AQs derivatives is mediated by miRNA regulation and by the orchestration of PI3K/Akt/mTOR pathway, abnormally activated in many tumorigenesis processes. Moreover, AQs trigger modification of the epigenetic state of cancer cells, nowadays recognized as a valuable anticancer strategy.

Some AQ compounds induce caspase-independent cell death mechanisms, such as necroptosis by emodin [68] and ferroptosis by physcion [94], prompting cytotoxic effects in tumor cells with impaired apoptotic pathway. Given the contribution of apoptosis resistance to the failure of chemotherapy, compounds triggering non-canonical cell deaths represent a new resource in the armamentarium to fight cancer.

Many AQs disclose distinct anticancer activity against different types of cancers, and this may represent an interesting asset, suggesting that the diversity of AQs may encounter the biological diversity of the tumors. As an example, the well-known AQ emodin exerts anticancer effects in several types of cancer and the mechanisms of action vary according to the type of cancer. Based on the analysis of in vitro and in vivo data, emodin emerges as a promising anticancer agent particularly in digestive system cancers, such as pancreatic cancer, where it modulates different angiogenesis-related miRNAs and blocks EMT and the formation of hepatic metastases [130].

Despite the considerable potential of AQs demonstrated in preclinical studies, some issues still need consideration before translating those results to clinical trials. For instance, the selectivity towards cancer cells compared to their non-transformed counterpart has only partially been addressed, showing contrasting evidence, ranking from good selectivity for aspergiolide A, bostrycin, and SZ-685C to the total absence of selectivity for G503.

Nevertheless, the most concerning issue about AQs is their genotoxic and carcinogenic potential. This review outlined the state of knowledge of the genetic toxicity of AQs and their metabolites. Notwithstanding more than 30 years of evidence, the recent evaluation of EFSA was unable to provide a daily intake of AQs that does not raise concerns about harmful effects for the general population, and in particular for the most vulnerable groups. The most alarming aspect is that genotoxic compounds are characterized by a dose-response curve that suggests the absence of a threshold to guarantee the absence of risk for human health compared to non-genotoxic compounds [183]. The precautionary conclusion is that AQs may be considered genotoxic and carcinogenic unless incoming specific data demonstrate the contrary [144]. Although many anticancer drugs target DNA to trigger cancer cell death, the toxicological profile of AQs has still to be clearly disclosed to guarantee an appropriate risk/benefit assessment, as with all therapeutic agents. The pharmacokinetics and the genotoxic profile of the different metabolites of AQs has to be assessed through in vitro and in vivo studies, including different and innovative approaches such as read-across and metabonomic studies already used for the well-known emodin [184,185]. Of note, the European Commission published a regulation entered into force on 7 April 2021 stating that aloe-emodin and all extracts in which this substance is present, emodin and all extracts in which this substance is present, and danthron and all extracts in which this substance is present have been added to Part A Annex III of Regulation (EC) No 1925/2006, meaning that they are prohibited for use in food [186]. This decision came following a scientific opinion from EFSA [144] that “hydroxyanthracene derivatives aloe-emodin, emodin and danthron (…) are genotoxic and can cause cancer in the intestine” [144].

To conclude, AQs and derivatives from marine microorganisms present outstanding potential as innovative anticancer drugs. However, the translation to clinical trials is still missing, and the definition of their toxicological profile is a necessary step before conceiving their clinical use as anticancer agents. 

## Figures and Tables

**Table 1:** Cytotoxic activity of anthraquinones isolated from marine-derived fungi.

Compound	Fungus Species	Source of Isolation	Cell Line(s)	IC_50_^a^/ Cell Growth Inhibition Rate	Time (Where Indicated)	Reference
(1’S)-6,1′-O,O-dimethylaverantin	*Aspergillus* sp. (strain SCSIO F063)	Marine sediment	SPF-268 MCF-7 NCI-H460	>50 μM >50 μM >50 μM	72 h 72 h 72 h	[26]
(1′S)-7-chloroaverantin	*Aspergillus* sp. (strain SCSIO F063)	Marine sediment	SPF-268 MCF-7 NCI-H460	>50 μM 36.41 μM >50 μM	72 h 72 h 72 h	[26]
(cis)-emodin-physcion bianthrone	*Aspergillus glaucus*	Marine sediment	HL-60 A549	44 μM 14.2 μM	/	[27]
(trans)-emodin-physcion bianthrone	*Aspergillus glaucus*	Marine sediment	HL-60 A549	7.8 μM 9.2 μM	/	[27]
1,2,3-trimethoxy-7-hydroxymethylanthracene-9,10-dione	*Aspergillus tritici* (strain SP2-8-1)	Coral *Galaxea fascicularis*	HeLa A549 HepG2	>50 μM >50 μM 42.07 μM	48 h 48 h 48 h	[28]
1,4,6- trihydroxy-2-methoxy-7-methylanthracene-9,10-dione	*Halorosellinia* sp. (strain 1403)	Mangrove *Kandelia candel* (L.) Druce	KB KBv200	>50 μg/mL >50 μg/mL	72 h 72 h	[29]
1-hydroxy-6-methyl-8-hydroxymethylxanthone	*Chaetomium* sp. (strain YMF 1.02105)	Submerged woody substrate	A549 Raji HepG2 MCF-7 HL-60	>100 μg/mL >100 μg/mL >100 μg/mL >100 μg/mL >100 μg/mL	/	[30]
1′-O-methyl-7-chloroaverantin	*Aspergillus* sp. (strain SCSIO F063)	Marine sediment	SPF-268 MCF-7 NCI-H460	34.06 μM 26.09 μM 37.19 μM	72 h 72 h 72 h	[26]
1′-O-methylaverantin	*Aspergillus* sp. (strain SCSIO F063)	Marine sediment	SPF-268 MCF-7 NCI-H460	33.59 μM 35.31 μM 44.22 μM	72 h 72 h 72 h	[26]
1403P-3	Endophytic fungus No. 1403 (strain 1403)	Mangrove (specie was not indicated)	KB KBv200	19.66 μM 19.27 μM	72 h 72 h	[31]
			MCF-7 MDA-MB-435	9.7 μM 7.6 μM	/	[32]
2-O-Acetylaltersolanol B	*Stemphylium* sp. (strain 33231)	Mangrove *Burguiera sexangula* var. *rhynchopetala*	B16F10 A549	>10 μM >10 μM	/	[33]
2-O-Acetylaltersolanol L	*Stemphylium* sp. (strain 33231)	Mangrove *Burguiera sexangula* var. *rhynchopetala*	B16F10 A549	>10 μM >10 μM	/	[33]
3-hydroxy-1,2,5,6-tetramethoxyanthracene-9,10-dione	*Aspergillus tritici* (strain SP2-8-1)	Coral *Galaxea fascicularis*	HeLa A549 HepG2	>50 μM >50 μM >50 μM	48 h 48 h 48 h	[28]
4R,8-dihydroxyconiothyrione B	*Talaromyces islandicus* (strain EN-501)	Red alga *Laurencia* *okamurai*	A2780 A2780 CisR	<10 μM <10 μM	/	[34]
4S,8-dihydroxyconiothyrinone B	*Talaromyces islandicus* (strain EN-501)	Red alga *Laurencia* *okamurai*	A2780 A2780 CisR	<10 μM <10 μM	/	[34]
4S,8-dihydroxy-10-O-methyldendryol E	*Talaromyces islandicus* (strain EN-501)	Red alga *Laurencia* *okamurai*	A2780 A2780 CisR	<10 μM <10 μM	/	[34]
6-O-methyl-averantin	*Aspergillus* sp. (strain SCSIO F063)	Marine sediment	SPF-268 MCF-7 NCI-H460	>50 μM >50 μM >50 μM	72 h 72 h 72 h	[26]
6-O-methyl-7-bromoaverantin	*Aspergillus* sp. (strain SCSIO F063)	Marine sediment	SPF-268 MCF-7 NCI-H460	24.69 μM 25.62 μM 18.91 μM	72 h 72 h 72 h	[26]
6-O-methyl-7-chloroaverantin	*Aspergillus* sp. (strain SCSIO F063)	Marine sediment	SPF-268 MCF-7 NCI-H460	7.11 μM 6.64 μM 7.42 μM	72 h 72 h 72 h	[26]
6-O-methyl-7-chloroaverythrin	*Aspergillus* sp. (strain SCSIO F063)	Marine sediment	SPF-268 MCF-7 NCI-H460	>50 μM 24.38 μM >50 μM	72 h 72 h 72 h	[26]
6,1′-O,O-dimethyl-7-bromoaverantin	*Aspergillus* sp. (strain SCSIO F063)	Marine sediment	SPF-268 MCF-7 NCI-H460	>50 μM >50 μM >50 μM	72 h 72 h 72 h	[26]
6,1′-O,O-dimethyl-7-chloroaverantin	*Aspergillus* sp. (strain SCSIO F063)	Marine sediment	SPF-268 MCF-7 NCI-H460	>50 μM >50 μM >50 μM	72 h 72 h 72 h	[26]
7-chloroaverantin-1′-butyl ether	*Aspergillus* sp. (strain SCSIO F063)	Marine sediment	SPF-268 MCF-7 NCI-H460	>50 μM 49.53 μM >50 μM	72 h 72 h 72 h	[26]
7-chloroaverythrin	*Aspergillus* sp. (strain SCSIO F063)	Marine sediment	SPF-268 MCF-7 NCI-H460	>50 μM >50 μM >50 μM	72 h 72 h 72 h	[26]
8- hydroxyconiothyrinone B	*Talaromyces islandicus* (strain EN-501)	Red alga *Laurencia* *okamurai*	A2780 A2780 CisR	<10 μM <10 μM	/	[34]
8-O-Methylversicolorin B	*Aspergillus versicolor* co-cultured with *Bacillus subtilis*	Sponge *Agelas oroides*	L5178Y	21.2 μM	72 h	[35]
8,11-Dihydroxyconiothyrinone B	*Talaromyces islandicus* (strain EN-501)	Red alga *Laurencia* *okamurai*	A2780 A2780 CisR	<10 μM <10 μM	/	[34]
9R-hydroxydihydrodesoxybostrycin	*Fusarium* spp. (strains PSU-F14 and PSU-F135)	Gorgonian sea fan (*Annella* sp.)	KB MCF-7 Vero ^b^	19 μM 15 μM 57 μM	/	[36]
9R-hydroxyhalorosellinia A	*Fusarium* spp. (strains PSU-F14 and PSU-F135)	Gorgonian sea fan (*Annella* sp.)	KB MCF-7 Vero ^b^	49 μM 6.2 μM 54 μM	/	[36]
Alterporriol A	*Stemphylium* sp. (strain 33231)	Mangrove *Burguiera sexangula* var. *rhynchopetala*	B16F10 A549	>10 μM >10 μM	/	[33]
Alterporriol B	*Stemphylium* sp. (strain 33231)	Mangrove *Burguiera sexangula* var. *rhynchopetala*	B16F10 A549	>10 μM >10 μM	/	[33]
Alterporriol C	*Stemphylium* sp. (strain 33231)	Mangrove *Burguiera sexangula* var. *rhynchopetala*	B16F10 A549	>10 μM >10 μM	/	[33]
	*Alternaria* sp. (strain ZJ-2008003)	Soft coral *Sarcophyton* sp.	HCT-116 PC-3 HepG2 Hep3B MCF-7/ADR	24 μM 27 μM 53 μM 51 μM 98 μM	/	[37]
Alterporriol D	*Stemphylium* sp. (strain 33231)	Mangrove *Burguiera sexangula* var. *rhynchopetala*	B16F10 A549	>10 μM >10 μM	/	[33]
Alterporriol E	*Stemphylium* sp. (strain 33231)	Mangrove *Burguiera sexangula* var. *rhynchopetala*	B16F10 A549	>10 μM >10 μM	/	[33]
Alterporriol K	*Alternaria* sp. (strain ZJ9-6B)	Fruit of mangrove *Aegiceras corniculatum*	MDA-MB-435 MCF-7	26.97 μM 29.11 μM	/	[38]
Alterporriol L	*Alternaria sp.* (strain ZJ9-6B)	Fruit of mangrove *Aegiceras corniculatum*	MDA-MB-435 MCF-7	13.11 μM 20.04 μM	/	[38]
	*Alternaria sp.* (strain ZJ9-6B)	Mangrove *Aegiceras corniculatum*	MCF-7 MDA-MB-435	20.04 μM 13.11 μM	48 h 48 h	[39]
Alterporriol N	*Alternaria* sp. (strain ZJ-2008003)	Soft coral *Sarcophyton* sp.	HCT-116 PC-3 HepG2 Hep3B MCF-7/ADR	>100 μM >100 μM >100 μM >100 μM >100 μM	/	[37]
Alterporriol O	*Alternaria* sp. (strain ZJ-2008003)	Soft coral *Sarcophyton* sp.	HCT-116 PC-3 HepG2 Hep3B MCF-7/ADR	>100 μM >100 μM >100 μM >100 μM >100 μM	/	[37]
Alterporriol P	*Alternaria* sp. (strain ZJ-2008003)	Soft coral *Sarcophyton* sp.	HCT-116 PC-3 HepG2 Hep3B MCF-7/ADR	8.6 μM 6.4 μM 20 μM 21 μM 23 μM	/	[37]
Alterporriol R	*Stemphylium* sp. (strain 33231)	Mangrove *Burguiera sexangula* var. *rhynchopetala*	B16F10 A549	>10 μM >10 μM	/	[33]
Alterporriol U	*Stemphylium* sp. (strain 33231)	Mangrove *Burguiera sexangula* var. *rhynchopetala*	B16F10 A549	>10 μM >10 μM	/	[33]
Alterporriol V	*Stemphylium* sp. (strain 33231)	Mangrove *Burguiera sexangula* var. *rhynchopetala*	B16F10 A549	>10 μM >10 μM	/	[33]
Alterporriol W	*Stemphylium* sp. (strain 33231)	Mangrove *Burguiera sexangula* var. *rhynchopetala*	B16F10 A549	>10 μM >10 μM	/	[33]
Alterporriol Y	*Stemphylium lycopersici*	Coral *Dichotella gemmacea*	HCT-116 MCF- Hu7	>50 μM >50 μM >50 μM	/	[40]
Altersolanol A	*Stemphylium lycopersici*	Coral *Dichotella gemmacea*	HCT-116 MCF-7 Hu7	1.3 μM 7.2 μM 38 μM	/	[40]
Altersolanol B	*Alternaria* sp. (strain ZJ-2008003)	Soft coral *Sarcophyton* sp.	HCT-116 PC-3 HepG2 Hep3B MCF-7/ADR	>100 μM >100 μM >100 μM >100 μM >100 μM	/	[37]
	*Stemphylium lycopersici*	Coral *Dichotella gemmacea*	HCT-116 MCF-7 Hu7	3.5 μM 9 μM >50 μM	/	[40]
	*Stemphylium* sp. (strain 33231)	Mangrove *Burguiera sexangula* var. *rhynchopetala*	B16F10 A549	>10 μM >10 μM	/	[33]
Altersolanol C	*Alternaria* sp. (strain ZJ-2008003)	Soft coral *Sarcophyton* sp.	HCT-116 PC-3 HepG2 Hep3B MCF-7/ADR	2.2 μM 7.6 μM 8.9 μM 8.2 μM 3.2 μM	/	[37]
Altersolanol L	*Alternaria* sp. (strain ZJ-2008003)	Soft coral *Sarcophyton* sp.	HCT-116 PC-3 HepG2 Hep3B MCF-7/ADR	>100 μM >100 μM >100 μM >100 μM >100 μM	/	[37]
	*Stemphylium* sp. (strain 33231)	Mangrove *Burguiera sexangula* var. *rhynchopetala*	B16F10 A549	>10 μM >10 μM	/	[33]
Ampelanol	*Alternaria* sp. (strain ZJ-2008003)	Soft coral )*Sarcophyton* sp.	HCT-116 PC-3 HepG2 Hep3B MCF-7/ADR	>100 μM >100 μM >100 μM >100 μM >100 μM	/	[37]
	*Stemphylium lycopersici*	Coral *Dichotella gemmacea*	HCT-116 MCF-7 Hu7	>50 μM >50 μM >50 μM	/	[40]
Aspergiolide A	*Aspergillus glaucus* (strain HB1-19)	Marine sediment	HeLa A549 K562 HL-60 HCT-116	3.1 μM 0.1 μM 7.1 μM 0.3 μM 4.4 μM	/	[41]
			P388 HL-60 BEL-7402 A549	35 μM 0.28 μM 7.5 μM 0.13 μM	/	[42]
			HeLa SMMC-7721 SGC-7901 MCF-7 MDA-MB-468 U251 A431 SK-OV-3 BxPC3 786-O Bel-7402	Range between 2.37 μM and 7.07 μM ^c^	72 h	[43]
Aspergiolide B	*Aspergillus glaucus*	Marine sediment	HL-60 A549	0.51 μM 0.24 μM	72 h 24 h	[27]
Aspergiolide C	*Aspergillus glaucus* (strain HB 1–19)	Marine sediment	P388 HL-60 BEL-7402 A549	>50 μM >50 μM >50 μM >50 μM	/	[44]
Aspergiolide D	*Aspergillus glaucus* (strain HB 1–19)	Marine sediment	P388 HL-60 BEL-7402 A549	>50 μM >50 μM >50 μM >50 μM	/	[44]
Aspetritone A (or 3, 9-deoxy-7-methoxybostrycin)	*Aspergillus tritici* (strain SP2-8-1)	Coral *Galaxea fascicularis*	HeLa A549 HepG2	2.67 μM 3.13 μM 3.87 μM	48 h 48 h 48 h	[28]
Aspetritone B (or 1,2,3,4-tetrahydro-2,3,5-trihydroxy-3-methyl-6,7-dimethoxyanthracene-9,10-dione)	*Aspergillus tritici* (strain SP2-8-1)	Coral *Galaxea fascicularis*	HeLa A549 HepG2	10.57 μM 4.67 μM 8.57 μM	48 h 48 h 48 h	[28]
Austrocortirubin	*Halorosellinia* sp. (strain 1403)	Mangrove *Kandelia candel* (L.) Druce	KB KBv200	>50 μg/mL >50 μg/mL	72 h 72 h	[29]
	*Fusarium* spp. (strain PSU- F14 and PSU-F135)	Sea fan *Annella* sp.	MCF-7	6.3 μM	/	[36]
Auxarthrol C	*Stemphylium lycopersici*	Coral *Dichotella gemmacea*	HCT-116 MCF-7 Hu7	>50 μM >50 μM >50 μM	/	[40]
	*Stemphylium* sp. (strain 33231)	Mangrove *Burguiera sexangula* var. *rhynchopetala*	B16F10 A549	>10 μM >10 μM	/	[33]
Auxarthrol D	*Sporendonema casei* (strain HDN16-802)	Marine sediment	HL-60 K562 HeLa HCT-116 MGC-803 HOS8910 MDA-MB-231 SH-SY5Y PC-3 BEL-7402 L-02 ^b^	7.5 μM >50 μM >50 μM 14.5 μM 21.8 μM >50 μM 19.1 μM 22.9 μM 21.9 μM 16.6 μM >50 μM	72 h 72 h 72 h 72 h 72 h 72 h 72 h 72 h 72 h 72 h 72 h	[45]
Auxarthrol F	*Sporendonema casei* (strain HDN16-802)	Marine sediment	HL-60 K562 HeLa HCT-116 MGC-803 HOS8910 MDA-MB-231 SH-SY5Y PC-3 BEL-7402 L-02 ^b^	4.5 μM 16.5 μM 10.7 μM 7.8 μM 17.7 μM 18.7 μM 10.1 μM 17.2 μM 20 μM 21.3 μM 22.2 μM	72 h 72 h 72 h 72 h 72 h 72 h 72 h 72 h 72 h 72 h 72 h	[45]
Averantin	*Aspergillus* sp. (strain SCSIO F063)	Marine sediment	SPF-268 MCF-7 NCI-H460	>50 μM 45.47 μM >50 μM	72 h 72 h 72 h	[26]
	*Penicillium flavidorsum* (strain SHK1-27)	Marine sediment	K562	27.7 μM	/	[46]
	*Aspergillus versicolor*	Sponge *Petrosia* sp.	A549 SKOV-3 SK-MEL-2 XF-498 HCT-15	3.15 μg/mL 3.88 μg/mL 3.57 μg/mL 3.04 μg/mL 3.13 μg/mL	72 h 72 h 72 h 72 h 72 h	[47]
Averantin-1′-butyl ether	*Aspergillus* sp. (strain SCSIO F063)	Marine sediment	SPF-268 MCF-7 NCI-H460	47.19 μM 40.47 μM >50 μM	72 h 72 h 72 h	[26]
Averufin	*Aspergillus versicolor*	Sponge *Petrosia* sp.	A549 SKOV-3 SK-MEL-2 XF-498 HCT-15	14.92 μg/mL 14.07 μg/mL 14.56 μg/mL 12.04 μg/mL 11.97 μg/mL	72 h 72 h 72 h 72 h 72 h	[47]
Averythrin	*Aspergillus* sp. (strain SCSIO F063)	Marine sediment	SPF-268 MCF-7 NCI-H460	>50 μM 29.69 μM >50 μM	72 h 72 h 72 h	[26]
Bostrycin	Not indicated	Not indicated	DU-145	25.31 μM 8.62 μM 4.79 μM	24 h 48 h 72 h	[48]
	Not indicated	Not indicated	MGC-803	29.7 μM 14.07 μM 13.47 μM	24 h 48 h 72 h	[49]
	*Nigrospora* sp. (strain 1403)	Mangrove *Kandelia candel* (L.) Druce	A549 Hep-2 HepG2 KB MCF-7 MCF-7/ADR	2.64 μg/mL 5.39 μg/mL 5.90 μg/mL 4.19 μg/mL 6.13 μg/mL 6.68 μg/mL	/	[50]
	*Fusarium* spp. (strain PSU- F14 and PSU-F135)	Sea fan *Annella* sp.	KB MCF-7 Vero ^b^	0.9 μM 2.7 μM 4.2 μM	/	[36]
Catenarin	*Sporidesmium* *circinophorum* (strain KUFA 0043)	Sponge *Petrosia* sp.	MCF-7 A375-C5 NCI-H460	>150 mM ^c^	/	[51]
Chrisophanic acid (or chrysophanol)	*Aspergillus candidus* (strain KUFA0062)	Sponge *Epipolasis* sp.	HepG2 HT-29 HCT-116 A549 A375 MCF-7 U251 T98G	88.9% (100 μM) ^d^ 98.7% (100 μM) ^d^ 101.7% (100 μM) ^d^ 98.6% (100 μM) ^d^ 89.5% (100 μM) ^d^ 108.1% (100 μM) ^d^ 102.7% (100 μM) ^d^ 112% (100 μM) ^d^	48 h 48 h 48 h 48 h 48 h 48 h 48 h 48 h	[52]
Chrysazin (or danthron)	*Beauveria bassiana* (strain TPU942)	Unidentified sponge	HCT-15 Jurkat	>30 μM >30 μM	48 h 48 h	[53]
Compound 6	*Halorosellinia* sp. (strain 1403) and *Guignardia* sp. (strain 4382)	Mangrove (specie was not indicated)	KB KBv200	3.17 μM 3.21 μM	72 h 72 h	[54]
Demethoxyaustrocortirubin	*Halorosellinia* sp. (strain 1403)	Mangrove *Kandelia candel* (L.) Druce	KB KBv200	>50 μg/mL >50 μg/mL	72 h 72 h	[29]
Deoxybostrycin	*Nigrospora* sp. (strain 1403)	Mangrove *Kandelia candel* (L.) Druce	A549 Hep-2 HepG2 KB MCF-7 MCF-7/ADR	2.44 μg/mL 3.15 μg/mL 4.41 μg/mL 3.15 μg/mL 4.76 μg/mL 5.46 μg/mL	/	[50]
Dihydroaltersolanol A	*Alternaria* sp. (strain ZJ-2008003)	Soft coral *Sarcophyton* sp.	HCT-116 PC-3 HepG2 Hep3B MCF-7/ADR	>100 μM >100 μM >100 μM >100 μM >100 μM	/	[37]
Emodin	*Aspergillus tritici* (strain SP2-8-1)	Coral *Galaxea fascicularis*	HeLa A549 HepG2	25.07 μM 22.17 μM 30.20 μM	48 h 48 h 48 h	[28]
	*Neosartorya fischeri* (strain 1008F1)	Marine-derived (source was not specified)	SGC7901 BEL-7404	32.7% (200 μg/mL) ^d^ 11.3% (200 μg/mL) ^d^	72 h 72 h	[55]
	*Penicillium* sp. (strain SCSIO41015)	Sponge *Callyspongia* sp.	MGC803	5.19 μM	/	[56]
Fusaquinon A	*Fusarium* sp. (strain ZH-210)	Mangrove sediment	KB KBv200 MCF-7	>50 μg/mL >50 μg/mL >50 μg/mL	/	[57]
Fusaquinon B	*Fusarium* sp. (strain ZH-210)	Mangrove sediment	KB KBv200 MCF-7	>50 μg/mL >50 μg/mL >50 μg/mL	/	[57]
Fusaquinon C	*Fusarium* sp. (strain ZH-210)	Mangrove sediment	KB KBv200 MCF-7	>50 μg/mL >50 μg/mL >50 μg/mL	/	[57]
Fusarnaphthoquinone A	*Fusarium* spp. (strain PSU- F14 and PSU-F135)	Sea fan *Annella* sp.	KB MCF-7	130 μM 22 μM	/	[36]
G503	*Nigrospora* sp. (strain 1403)	Mangrove *Kandelia candel* (L.) Druce	HONE-1 CNE2 5–8F A549 HepG2 B7402 Rb PC-3 SGC7901 HUVEC ^b^ Chang liver cells ^b^	35.7 μM 35.6 μM 31.7 μM 20 μM 13.3 μM 19.8 μM 44 μM 21.1 μM 10.24 μM 22.4 μM 17.5 μM	48 h 48 h 48 h 48 h 48 h 48 h 48 h 48 h 48 h 48 h 48 h 48 h	[58]
Halorosellinia A (1,4,5,6,7,9-hexahydroxy-2-methoxy-7α-methyl-5β,9β,8αβ,6α,10aα- hexahydroanthracen-10(10aH)-one)	*Halorosellinia* sp. (strain 1403)	Mangrove *Kandelia candel* (L.) Druce	KB KBv200	>50 μg/mL >50 μg/mL	72 h 72 h	[29]
Hydroxy-9,10-anthraquinone	*Halorosellinia* sp. (strain 1403)	Mangrove *Kandelia candel* (L.) Druce	KB KBv200	1.40 μg/mL 2.58 μg/mL	72 h 72 h	[29]
Macrosporin	*Stemphylium lycopersici*	Coral *Dichotella gemmacea*	HCT-116 MCF-7 Hu7	>50 μM >50 μM >50 μM	/	[40]
Macrosporin 2-O-(6′-acetyl)-a-D-glucopyranoside	*Stemphylium* sp. (strain 33231)	Mangrove *Burguiera sexangula* var. *rhynchopetala*	B16F10 A549	>10 μM >10 μM	/	[33]
Macrosporin 2-O-a-D-glucopyranoside	*Stemphylium lycopersici*	Coral *Dichotella gemmacea*	HCT-116 MCF-7 Hu7	>50 μM >50 μM >50 μM	/	[40]
Methyl-averantin	*Aspergillus versicolor*	Sponge *Petrosia* sp.	A549 SKOV-3 SK-MEL-2 XF-498 HCT-15	0.64 μg/mL 1.17 μg/mL 1.10 μg/mL 0.41 μg/mL 0.49 μg/mL	72 h 72 h 72 h 72 h 72 h	[47]
Nidurufin	*Aspergillus versicolor*	Sponge *Petrosia* sp.	A549 SKOV-3 SK-MEL-2 XF-498 HCT-15	1.83 μg/mL 3.39 μg/mL 3.16 μg/mL 1.78 μg/mL 2.20 μg/mL	72 h 72 h 72 h 72 h 72 h	[47]
	*Aspergillus versicolor* (strain A-21-2-7)	Marine sediment	A549	25.97 μM	/	[59]
	*Penicillium flavidorsum* (strain SHK1-27)	Marine sediment	K562	12.6 μM	24 h	[46]
Nigrosporin B	*Fusarium* spp. (strain PSU- F14 and PSU-F135)	Sea fan *Annella* sp.	KB MCF-7 Vero ^b^	88 μM 5.4 μM 29 μM	/	[36]
Norsolorinic acid	*Aspergillus nidulans*	Not specified	MCF-7	12.7 μM	48 h	[60]
Penicillanthramin A	*Penicillium citrinum* (strain PSU-F51)	Sea fan *Annella* sp.	KB	30 μg/mL	/	[61]
Physcion	*Sporidesmium* *circinophorum* (strain KUFA 0043)	Sponge *Petrosia* sp.	MCF-7 A375-C5 NCI-H460	>150 mM (GI_50_^e^)	/	[51]
	*Microsporum* sp. (Strain MFS-YL)	Red alga *Lomentaria catenata*	HeLa	/		[62]
SZ-685C	*Halorosellinia* sp. (strain 1403)	Mangrove *Kandelia candel* (L.) Druce	NFPA MMQ RPCs ^b^	18.76 μM 14.51 μM 56.09 μM	24 h 24 h 24 h	[63]
	*Halorosellinia* sp. (strain 1403)	Mangrove *Kandelia candel* (L.) Druce	MMQ RPCs ^b^	13.2 μM 49.1 μM	48 h 48 h	[64]
	*Halorosellinia* sp. (strain 1403)	Mangrove *Kandelia candel* (L.) Druce	CNE2	46.89 μM 14.13 μM 8.97 μM	24 h 48 h 72 h	[65]
			CNE2R	69.11 μM 17.86 μM 8.94 μM	24 h 48 h 72 h	
	*Halorosellinia* sp. (strain 1403)	Mangrove *Kandelia candel* (L.) Druce	MCF-7 MCF-7/ADR MCF-7/Akt K562 K562/ADR HL-60 HL-60/ADR	7.38 μM 4.17 μM 3.36 μM 1.09 μM 1.35 μM 1.94 μM 1.76 μM	48 h 48 h 48 h 48 h 48 h 48 h 48 h	[66]
	*Halorosellinia* sp. (strain 1403)	Mangrove *Kandelia candel* (L.) Druce	MCF-7 MDA-MB-435	7.5 μM 3 μM	48 h 48 h	[67]
Tetrahydroaltersolanol B	*Alternaria* sp. (strain ZJ-2008003)	Soft coral *Sarcophyton* sp.	HCT-116 PC-3 HepG2 Hep3B MCF-7/ADR	>100 μM >100 μM >100 μM >100 μM >100 μM	/	[37]
Tetrahydroaltersolanol C	*Alternaria* sp. (strain ZJ-2008003)	Soft coral *Sarcophyton* sp.	HCT-116 PC-3 HepG2 Hep3B MCF-7/ADR	>100 μM >100 μM >100 μM >100 μM >100 μM	/	[37]
Tetrahydroaltersolanol D	*Alternaria* sp. (strain ZJ-2008003)	Soft coral *Sarcophyton* sp.	HCT-116 PC-3 HepG2 Hep3B MCF-7/ADR	>100 μM >100 μM >100 μM >100 μM >100 μM	/	[37]
Tetrahydroaltersolanol E	*Alternaria* sp. (strain ZJ-2008003)	Soft coral *Sarcophyton* sp.	HCT-116 PC-3 HepG2 Hep3B MCF-7/ADR	>100 μM >100 μM >100 μM >100 μM >100 μM	/	[37]
Tetrahydroaltersolanol F	*Alternaria* sp. (strain ZJ-2008003)	Soft coral *Sarcophyton* sp.	HCT-116 PC-3 HepG2 Hep3B MCF-7/ADR	>100 μM >100 μM >100 μM >100 μM >100 μM	/	[37]
Versicolorin B	*Aspergillus versicolor* (strain A-21-2-7)	Marine sediment	A549 A2780	25.60 μM 38.76 μM	/	[59]
Versiconol	*Aspergillus versicolor*	Sponge *Petrosia* sp.	A549 SKOV-3 SK-MEL-2 XF-498 HCT-15	20.45 μg/mL 15.29 μg/mL 15.86 μg/mL 23.73 μg/mL 19.02 μg/mL	72 h 72 h 72 h 72 h 72 h	[47]

Abbreviations. ^a^: concentration which produces half of the maximum viability reduction; ^b^: non-tumorigenic cells; ^c^: single IC50 values were not indicated; ^d^: percentage of cell viability relative to control (100%); ^e^: GI50: concentration that inhibits 50% of cell growth); A2780 CisR: A2780 cells resistant to cisplatin; CNE2R: radioresistant CNE2 cell line; HL-60/ADR: HL-60 cells resistant to adriamycin; K562/ADR: K562 cells resistant to adriamycin; KBv200: multidrug resistant KB cells; MCF-7/ADR: MCF-7 cells resistant to adriamycin; MCF-7/Akt: MCF-7 cells that constitutively express active Akt (protein kinase B); NFPA: nonfunctioning pituitary adenoma; RPCs: normal pituitary cells; sp.: species.

**Table 2:** Cytotoxic activity of anthraquinones isolated from other marine sources.

Compound	Chemical Class	Species	Source of Isolation	Cell Line(s)	IC_50_^a^/ Cell Growth Inhibition Rate	Time (Where Indicated)	Reference
1,8-dihydroxy-2-ethyl-3-methylanthraquinone	Anthraquinone	*Streptomyces* sp. (strain FX-58)	Marine plant *Salicornia herbacea*	HL-60 BGC-823 MDA-MB-435	6.83 μg/mL 82.2 μg/mL 56.59 μg/mL	/	[115]
3-hydroxy-1-keto-3-methyl-8- methoxy-1,2,3,4-tetrahydro-benz[α]anthracene	Anthraquinone	*Streptomyces* sp. (strain W007)	Marine sediment	BEL-7402 A549	37.5% (100 μM) ^b^ 65.5% (100 μM) ^b^	72 h 72 h	[116]
A-7884	*Angucycline glycoside*	*Streptomyces lusitanus* (SCSIO LR32)	Deep sea sediment	MDA-MB-435 MDA-MB-231 NCI-H460 HCT-116 HepG2 MCF10A ^c^	2.14 μM 4.80 μM 6.90 μM 0.48 μM 4.57 μM 2.68 μM	48 h	[117]
Dehydroxyaquayamycin	*Angucycline glycoside*	*Streptomyces* sp. (strain SCSIO11594)	Deep sea sediment	A549 CNE2 MCF-7 HepG2 HL7702 ^c^	16.40 μM 22.27 μM 23.65 μM 18.81 μM 49.34 μM	/	[118]
Deoxyrhodoptilometrin	Anthraquinone	/	Crinoid *Colobometra perspinosa*	SF-268 MCF-7 H460	72 μM 20 μM 25 μM	/	[112]
			Crinoid *Comanthus* sp.	C6 HCT-116	23.2 μM 13.1 μM	24 h 24 h	[113]
Fridamycin D	Angucycline glycoside	*Streptomyces* sp. (strain OC1610.4)	Deep sea sediment	MCF-7 MDA-MB-231 BT-474	7.58 μM 8.01 μM 6.46 μM	/	[119]
Galtamycin C	Angucycline glycoside	*Streptomyces* sp. (strain OC1610.4)	Deep sea sediment	L-O2 ^c^ HepG2 SMMC-7721 Plc-prf-5	>40 μM >40 μM >40 μM >40 μM	/	[120]
Galvaquinone A	Anthraquinone	*Streptomyces spinoverrucosus*	Marine sediment	Calu-3 H2887	>50 μM >50 μM	96 h 96 h	[121]
Galvaquinone B	Anthraquinone	*Streptomyces spinoverrucosus*	Marine sediment	Calu-3 H2887	12.2 μM 5 μM	96 h 96 h	[121]
Galvaquinone C	Anthraquinone	*Streptomyces spinoverrucosus*	Marine sediment	Calu-3 H2887	>50 μM >50 μM	96 h 96 h	[121]
Grincamycin	Angucycline glycoside	*Streptomyces lusitanus* (SCSIO LR32)	Deep sea sediment	B16 HepG2 SW-1990 HeLa NCI-H460 MCF-7	1.1 μM 5.3 μM 6.4 μM 5.3 μM 11 μM 2.1 μM	48 h 48 h 48 h 48 h 48 h 48 h	[122]
Grincamycin B	Angucycline glycoside	*Streptomyces lusitanus* (SCSIO LR32)	Deep sea sediment	B16 HepG2 SW-1990 HeLa NCI-H460 MCF-7	2.1 μM 8.5 μM 11 μM 6.4 μM >100 μM 12 μM	48 h 48 h 48 h 48 h 48 h 48 h	[122]
Grincamycin C	Angucycline glycoside	*Streptomyces lusitanus* (SCSIO LR32)	Deep sea sediment	HepG2 SW-1990 MCF-7	31 μM 31 μM 11 μM	48 h 48 h 48 h	[122]
Grincamycin D	Angucycline glycoside	*Streptomyces lusitanus* (SCSIO LR32)	Deep sea sediment	B16 HepG2 SW-1990 HeLa NCI-H460 MCF-7	9.7 μM 9.7 μM 22 μM 12 μM 30 μM 6.1 μM	48 h 48 h 48 h 48 h 48 h 48 h	[122]
Grincamycin E	Angucycline glycoside	*Streptomyces lusitanus* (SCSIO LR32)	Deep sea sediment	B16 HepG2 SW-1990 HeLa MCF-7	5.4 μM 11 μM 16 μM 11 μM 8.7 μM	48 h 48 h 48 h 48 h 48 h	[122]
Grincamycin F	Angucycline glycoside	*Streptomyces lusitanus* (SCSIO LR32)	Deep sea sediment	MCF-7	19 μM	48 h	[122]
Grincamycin G	Angucycline glycoside	*Streptomyces lusitanus* (SCSIO LR32)	Deep sea sediment	Jurkat	0.3 μM	72 h	[123]
Grincamycin H	Angucycline glycoside	*Streptomyces lusitanus* (SCSIO LR32)	Deep sea sediment	Jurkat	>20 μM	72 h	[123]
Grincamycin I	Angucycline glycoside	*Streptomyces lusitanus* (SCSIO LR32)	Deep sea sediment	MDA-MB-435 MDA-MB-231 NCI-H460 HCT-116 HepG2 MCF10A ^c^	10.20 μM 25.87 μM 11.87 μM 8.97 μM 9.41 μM 2.90 μM	48 h 48 h 48 h 48 h 48 h 48 h	[117]
Grincamycin J	Angucycline glycoside	*Streptomyces lusitanus* (SCSIO LR32)	Deep sea sediment	MDA-MB-435 MDA-MB-231 NCI-H460 HCT-116 HepG2 MCF10A ^c^	2.63 μM 4.68 μM 5.40 μM 2.63 μM 4.80 μM 2.43 μM	48 h 48 h 48 h 48 h 48 h 48 h	[117]
Grincamycin K	Angucycline glycoside	*Streptomyces lusitanus* (SCSIO LR32)	Deep sea sediment	MDA-MB-435 MDA-MB-231 NCI-H460 HCT-116 HepG2 MCF10A ^c^	>50 μM >50 μM >50 μM >50 μM >50 μM >50 μM	48 h 48 h 48 h 48 h 48 h 48 h	[117]
Grincamycin L	Angucycline glycoside	*Streptomyces lusitanus* (SCSIO LR32)	Deep sea sediment	MCF-7 MDA-MB-231 BT-474	>20 μM >20 μM >20 μM	/	[119].
Islandicin	Anthraquinone	*Streptomyces spinoverrucosus*	Marine sediment	Calu-3 H2887	>50 μM >50 μM	96 h 96 h	[121]
Kyamycin	Angucyclinone	*Streptomyces* sp. (strain M268)	Marine sediment	HL-60 A549 BEL-7402	68.8% (100 μM) 55.9% (100 μM) 31.7% (100 μM)	72 h 72 h 72 h	[124]
Landomycin N	Angucycline glycoside	*Streptomyces* sp. (strain OC1610.4)	Deep sea sediment	L-02 ^c^ HepG2 SMMC-7721 Plc-prf-5	>40 μM >40 μM >40 μM >40 μM	/	[120]
Lupinacidin A	Anthraquinone	*Streptomyces spinoverrucosus*	Marine sediment	Calu-3 H2887	3.1 μM 8.8 μM	96 h 96 h	[121]
Marangucycline A	Angucycline glycoside	*Streptomyces* sp. (strain SCSIO11594)	Deep sea sediment	A549 CNE2 MCF-7 HepG2 HL7702 ^c^	>50 μM >50 μM >50 μM >50 μM >50 μM	/	[118]
Marangucycline B	Angucycline glycoside	*Streptomyces* sp. (strain SCSIO11594)	Deep sea sediment	A549 CNE2 MCF-7 HepG2 HL7702 ^c^	0.45 μM 0.56 μM 0.24 μM 0.43 μM 3.67 μM	/	[118]
Marmycin A	Angucycline glycoside	*Streptomyces* sp. (strain CHN990)	Marine sediment	HCT-116	60.5 nM	72 h	[125]
Marmycin B	Angucycline glycoside	*Streptomyces* sp. (strain CHN990)	Marine sediment	HCT-116	1.09 μM	72 h	[125]
Moromycin B	Angucycline glycoside	*Streptomyces* sp. (strain OC1610.4)	Deep sea sediment	MCF-7 MDA-MB-231 BT-474	0.42 μM 0.35 μM 0.67 μM	/	[119]
Resistomycin (or heliomycin)	Anthraquinone	*Actinobacterium Streptomyces* (strain AUBN1/7)	Marine sediment	HMO2 HepG2	0.005 μg/mL 0.008 μg/mL	48 h 48 h	[126]
Rhodocomatulin 5,7-dimethyl ether	Anthraquinone	/	-Sponge *Clathria (Thalysias) hirsuta* Hooper and Levi -Crinoid *Comatula* (*Validia*) *rotalaria* Lamarck	MCF-7	9% (10 μM) ^b^	72 h	[114]
Rhodoptilometrin	Anthraquinone	/	Crinoid *Colobometra perspinosa*	SF-268 MCF-7 H460	41 μM 21 μM 25 μM	/	[112]
			Crinoid *Chomanthus* sp.	C6 HCT-116	30 μM 40.1 μM	24 h 24 h	[113]
Saliniquinone A	Anthraquinone	*Actinomycete Salinispora arenicola* (Strain CNS-325)	Marine sediment	HCT-116	9.9 nM	72 h	[127]
Saquayamycin B	Angucycline glycoside	*Streptomyces lusitanus* (SCSIO LR32)	Deep sea sediment	Jurkat	37 nM	72 h	[123]
		*Streptomyces* sp. (strain OC1610.4)	Deep sea sediment	MCF-7 MDA-MB-231 BT-474	0.40 μM 0.38 μM 0.41 μM	/	[119].
		*Streptomyces* sp. (strain OC1610.4)	Deep sea sediment	L-02 ^c^ HepG2 SMMC-7721 Plc-prf-5	0.34 μM 0.14 μM 0.03 μM 0.24 μM	/	[120]
Saquayamycin B1	Angucycline glycoside	*Streptomyces* sp. (strain OC1610.4)	Deep sea sediment	MCF-7 MDA-MB-231 BT-474	0.24 μM 0.16 μM 0.28 μM	/	[119]
Strepnoneside A	Anthraquinone glycoside	*Streptomyces coelicolor* (strain WBF-16)	Marine sediment	HCT116	30.2 μM	48 h	[128]
Strepnoneside B	Anthraquinone glycoside	*Streptomyces coelicolor* (strain WBF-16)	Marine sediment	HCT116	40.2 μM	48 h	[128]
Tetracenomycin D	Anthraquinone	*Streptomyces corchorusii* (strain AUBN1/7)	Marine sediment	HMO2 HepG2	0.009 μg/mL 0.013 μg/mL	48 h 48 h	[126]
Vineomycin A1	Angucycline glycoside	*Streptomyces lusitanus* (SCSIO LR32)	Deep sea sediment	Jurkat	11 nM	72 h	[123]
Vineomycin B2	Angucycline glycoside	*Streptomyces lusitanus* (SCSIO LR32)	Deep sea sediment	Jurkat	0.3 μM	72 h	[123]
Vineomycin E	Angucycline glycoside	*Streptomyces* sp. (strain OC1610.4)	Deep sea sediment	MCF-7 MDA-MB-231 BT-474	6.07 μM 7.72 μM 4.27 μM	/	[119]
Vineomycin F	Angucycline glycoside	*Streptomyces* sp. (strain OC1610.4)	Deep sea sediment	MCF-7 MDA-MB-231 BT-474	>20 μM >20 μM >20 μM	/	[119]
Vineomycinone B2	Angucycline glycoside	*Streptomyces* sp. (strain OC1610.4)	Deep sea sediment	MCF-7 MDA-MB-231 BT-474	>20 μM >20 μM >20 μM	/	[119]

Abbreviations. ^a^: concentration which produces half of the maximum viability reduction; ^b^: percentage of cell viability relative to control (100%); ^c^ non-tumorigenic cells; sp.: species.

## Data Availability

Not applicable.

## References

[1] IARC–International Agency for Research on Cancer. https://www.iarc.who.int/.

[2] Appeltans W., Ahyong S.T., Anderson G., Angel M.V., Artois T., Bailly N., Bamber R., Barber A., Bartsch I., Berta A. (2012). The Magnitude of Global Marine Species Diversity. Curr. Biol..

[3] Khalifa S.A.M., Elias N., Farag M.A., Chen L., Saeed A., Hegazy M.-E.F., Moustafa M.S., Abd El-Wahed A., Al-Mousawi S.M., Musharraf S.G. (2019). Marine Natural Products: A Source of Novel Anticancer Drugs. Mar. Drugs.

[4] Dyshlovoy S.A., Honecker F. (2020). Marine Compounds and Cancer: Updates 2020. Mar. Drugs.

[5] Fouillaud M., Venkatachalam M., Girard-Valenciennes E., Caro Y., Dufossé L. (2016). Anthraquinones and Derivatives from Marine-Derived Fungi: Structural Diversity and Selected Biological Activities. Mar. Drugs.

[6] Minotti G., Menna P., Salvatorelli E., Cairo G., Gianni L. (2004). Anthracyclines: Molecular Advances and Pharmacologic Developments in Antitumor Activity and Cardiotoxicity. Pharmacol. Rev..

[7] Diaz-Muñoz G., Miranda I.L., Sartori S.K., de Rezende D.C., Diaz M.A.N. (2018). Anthraquinones: An overview. Studies in Natural Products Chemistry.

[8] Malik E.M., Müller C.E. (2016). Anthraquinones as Pharmacological Tools and Drugs. Med. Res. Rev..

[9] Wuthi-udomlert M., Kupittayanant P., Gritsanapan W. (2010). In Vitro Evaluation of Antifungal Activity of Anthraquione Derivatives of *Senna Alata*. J. Health Res..

[10] Malmir M., Serrano R., Silva O., Mendez-Vilas A. (2017). Anthraquinones as potential antimicrobial agents-a review. Antimicrobial Research: Novel Bioknowledge and Educational Programs.

[11] Osman C.P., Ismail N.H. (2018). Antiplasmodial Anthraquinones from Medicinal Plants: The Chemistry and Possible Mode of Actions. Nat. Prod. Commun..

[12] Chien S.-C., Wu Y.-C., Chen Z.-W., Yang W.-C. (2015). Naturally Occurring Anthraquinones: Chemistry and Therapeutic Potential in Autoimmune Diabetes. Evid-Based Compl. Alt..

[13] Kshirsagar A.D., Panchal P.V., Harle U.N., Nanda R.K., Shaikh H.M. (2014). Anti-Inflammatory and Antiarthritic Activity of Anthraquinone Derivatives in Rodents. Int. J. Inflam..

[14] Wu C.-M., Wu S.-C., Chung W.-J., Lin H.-C., Chen K.-T., Chen Y.-C., Hsu M.-F., Yang J.-M., Wang J.-P., Lin C.-N. (2007). Antiplatelet Effect and Selective Binding to Cyclooxygenase (COX) by Molecular Docking Analysis of Flavonoids and Lignans. Int. J. Mol. Sci..

[15] Seo E.J., Ngoc T.M., Lee S.-M., Kim Y.S., Jung Y.-S. (2012). Chrysophanol-8-O-Glucoside, an Anthraquinone Derivative in Rhubarb, Has Antiplatelet and Anticoagulant Activities. J. Pharmacol. Sci..

[16] Jackson T.C., Verrier J.D., Kochanek P.M. (2013). Anthraquinone-2-Sulfonic Acid (AQ2S) Is a Novel Neurotherapeutic Agent. Cell Death Dis..

[17] Tian W., Wang C., Li D., Hou H. (2020). Novel Anthraquinone Compounds as Anticancer Agents and Their Potential Mechanism. Future Med. Chem..

[18] Srinivas G., Babykutty S., Sathiadevan P.P., Srinivas P. (2007). Molecular Mechanism of Emodin Action: Transition from Laxative Ingredient to an Antitumor Agent. Med. Res. Rev..

[19] Huang Q., Lu G., Shen H.-M., Chung M.C.M., Ong C.N. (2007). Anti-Cancer Properties of Anthraquinones from Rhubarb. Med. Res. Rev..

[20] Kohlmeyer J., Kohlmeyer E. (1979). Marine Mycology.

[21] Pang K.-L., Overy D.P., Jones E.B.G., da Luz Calado M., Burgaud G., Walker A.K., Johnson J.A., Kerr R.G., Cha H.-J., Bills G.F. (2016). ‘Marine Fungi’ and ‘Marine-Derived Fungi’ in Natural Product Chemistry Research: Toward a New Consensual Definition. Fungal Biol. Rev..

[22] Rateb M.E., Ebel R. (2011). Secondary Metabolites of Fungi from Marine Habitats. Nat. Prod. Rep..

[23] Richards T.A., Jones M.D.M., Leonard G., Bass D. (2012). Marine Fungi: Their Ecology and Molecular Diversity. Ann. Rev. Mar. Sci..

[24] Liu L., Zheng Y.-Y., Shao C.-L., Wang C.-Y. (2019). Metabolites from Marine Invertebrates and Their Symbiotic Microorganisms: Molecular Diversity Discovery, Mining, and Application. Mar. Life Sci. Technol..

[25] Gessler N.N., Egorova A.S., Belozerskaya T.A. (2013). Fungal Anthraquinones. Appl. Biochem. Microbiol..

[26] Huang H., Wang F., Luo M., Chen Y., Song Y., Zhang W., Zhang S., Ju J. (2012). Halogenated Anthraquinones from the Marine-Derived Fungus *Aspergillus* Sp. SCSIO F063. J. Nat. Prod..

[27] Du L., Zhu T., Liu H., Fang Y., Zhu W., Gu Q. (2008). Cytotoxic Polyketides from a Marine-Derived Fungus *Aspergillus Glaucus*. J. Nat. Prod..

[28] Wang W., Liao Y., Tang C., Huang X., Luo Z., Chen J., Cai P. (2017). Cytotoxic and Antibacterial Compounds from the Coral-Derived Fungus *Aspergillus Tritici* SP2-8-1. Mar. Drugs.

[29] Xia X.-K., Huang H.-R., She Z.-G., Shao C.-L., Liu F., Cai X.-L., Vrijmoed L.L.P., Lin Y.-C. (2007). 1H And13C NMR Assignments for Five Anthraquinones from the Mangrove Endophytic Fungus *Halorosellinia* Sp. (No. 1403). Magn. Reson. Chem..

[30] Shen K.-Z., Gao S., Gao Y.-X., Wang A.-R., Xu Y.-B., Sun R., Hu P.-G., Yang G.-F., Li A.-J., Zhong D. (2012). Novel Dibenzo[b,e]Oxepinones from the Freshwater-Derived Fungus *Chaetomium* Sp. YMF 1.02105. Planta Med..

[31] Zhang J., Wu H., Xia X., Liang Y., Yan Y., She Z., Lin Y., Fu L. (2007). Anthracenedione Derivative 1403P-3 Induces Apoptosis in KB and KBv200 Cells via Reactive Oxygen Species-Independent Mitochondrial Pathway and Death Receptor Pathway. Cancer Biol. Ther..

[32] Yuan J., He Z., Wu J., Lin Y., Zhu X. (2011). A Novel Adriamycin Analogue Derived from Marine Microbes Induces Apoptosis by Blocking Akt Activation in Human Breast Cancer Cells. Mol. Med. Rep..

[33] Zhou X.-M., Zheng C.-J., Chen G.-Y., Song X.-P., Han C.-R., Li G.-N., Fu Y.-H., Chen W.-H., Niu Z.-G. (2014). Bioactive Anthraquinone Derivatives from the Mangrove-Derived Fungus *Stemphylium* Sp. 33231. J. Nat. Prod..

[34] Li H.-L., Li X.-M., Li X., Wang C.-Y., Liu H., Kassack M.U., Meng L.-H., Wang B.-G. (2017). Antioxidant Hydroanthraquinones from the Marine Algal-Derived Endophytic Fungus *Talaromyces Islandicus* EN-501. J. Nat. Prod..

[35] Abdel-Wahab N., Scharf S., Özkaya F., Kurtán T., Mándi A., Fouad M., Kamel M., Müller W., Kalscheuer R., Lin W. (2019). Induction of Secondary Metabolites from the Marine-Derived Fungus *Aspergillus Versicolor* through Co-Cultivation with *Bacillus Subtilis*. Planta Med..

[36] Trisuwan K., Khamthong N., Rukachaisirikul V., Phongpaichit S., Preedanon S., Sakayaroj J. (2010). Anthraquinone, Cyclopentanone, and Naphthoquinone Derivatives from the Sea Fan-Derived Fungi *Fusarium* Spp. PSU-F14 and PSU-F135. J. Nat. Prod..

[37] Zheng C.-J., Shao C.-L., Guo Z.-Y., Chen J.-F., Deng D.-S., Yang K.-L., Chen Y.-Y., Fu X.-M., She Z.-G., Lin Y.-C. (2012). Bioactive Hydroanthraquinones and Anthraquinone Dimers from a Soft Coral-Derived *Alternaria* Sp. Fungus. J. Nat. Prod..

[38] Huang C.-H., Pan J.-H., Chen B., Yu M., Huang H.-B., Zhu X., Lu Y.-J., She Z.-G., Lin Y.-C. (2011). Three Bianthraquinone Derivatives from the Mangrove Endophytic Fungus *Alternaria* Sp. ZJ9-6B from the South China Sea. Mar. Drugs.

[39] Huang C., Jin H., Song B., Zhu X., Zhao H., Cai J., Lu Y., Chen B., Lin Y. (2012). The Cytotoxicity and Anticancer Mechanisms of Alterporriol L, a Marine Bianthraquinone, against MCF-7 Human Breast Cancer Cells. Appl. Microbiol. Biotechnol..

[40] Li J., Zheng Y.-B., Kurtán T., Liu M.-X., Tang H., Zhuang C.-L., Zhang W. (2020). Anthraquinone Derivatives from a Coral Associated Fungus *Stemphylium Lycopersici*. Nat. Prod. Res..

[41] Qiao L., Duan Z., Chen Y., Luan Y., Gu Q., Liu Y.-K., Li D. (2019). Aspergiolides A and B: Core Structural Establishment and Synthesis of Structural Analogues. J. Org. Chem..

[42] Du L., Zhu T., Fang Y., Liu H., Gu Q., Zhu W. (2007). Aspergiolide A, a Novel Anthraquinone Derivative with Naphtho[1,2,3-de]Chromene-2,7-Dione Skeleton Isolated from a Marine-Derived Fungus *Aspergillus Glaucus*. Tetrahedron.

[43] Li J., Wang Y., Qi X., Li D., Zhu T., Mo X. (2014). Anticancer Efficacy and Absorption, Distribution, Metabolism, and Toxicity Studies of Aspergiolide A in Early Drug Development. Drug Des. Devel. Ther..

[44] Du L., Ai J., Li D., Zhu T., Wang Y., Knauer M., Bruhn T., Liu H., Geng M., Gu Q. (2011). Aspergiolides C and D: Spirocyclic Aromatic Polyketides with Potent Protein Kinase c-Met Inhibitory Effects. Chem. Eur. J..

[45] Ge X., Sun C., Feng Y., Wang L., Peng J., Che Q., Gu Q., Zhu T., Li D., Zhang G. (2019). Anthraquinone Derivatives from a Marine-Derived Fungus Sporendonema Casei HDN16-802. Mar. Drugs.

[46] Ren H., Liu W. (2011). Nidurufin as a New Cell Cycle Inhibitor from Marine-Derived Fungus *Penicillium Flavidorsum* SHK1-27. Arch. Pharm. Res..

[47] Lee Y.M., Li H., Hong J., Cho H.Y., Bae K.S., Kim M.A., Kim D.-K., Jung J.H. (2010). Bioactive Metabolites from the Sponge-Derived Fungus *Aspergillus Versicolor*. Arch. Pharm. Res..

[48] Lin W., Fang L.K., Liu J.W., Cheng W.Q., Yun M. (2008). Effect of Marine Fungal Metabolites from the South China Sea on Prostate Cancer Cell Line DU-145. J. Intern. Med..

[49] Chen C.Q., Fang L.K., Liu J.W. (2010). Effects of Marine Fungal Metabolites 1386A from the South China Sea on Proliferation, Apoptosis and Mitochondrial Membrane Potential in Gastric Cancer Cell Line MCG-803. Chin. J. Pathophys.

[50] Xia X., Li Q., Li J., Shao C., Zhang J., Zhang Y., Liu X., Lin Y., Liu C., She Z. (2011). Two New Derivatives of Griseofulvin from the Mangrove Endophytic Fungus *Nigrospora* Sp. (Strain No. 1403) from *Kandelia Candel* (L.) Druce. Planta Med..

[51] Buttachon S., May Zin W., Dethoup T., Gales L., Pereira J., Silva A., Kijjoa A. (2016). Secondary Metabolites from the Culture of the Marine Sponge-Associated Fungi *Talaromyces Tratensis* and *Sporidesmium Circinophorum*. Planta Med..

[52] Buttachon S., Ramos A.A., Inácio Â., Dethoup T., Gales L., Lee M., Costa P.M., Silva A.M.S., Sekeroglu N., Rocha E. (2018). Bis-Indolyl Benzenoids, Hydroxypyrrolidine Derivatives and Other Constituents from Cultures of the Marine Sponge-Associated Fungus *Aspergillus Candidus* KUFA0062. Mar. Drugs.

[53] Yamazaki H., Rotinsulu H., Kaneko T., Murakami K., Fujiwara H., Ukai K., Namikoshi M. (2012). A New Dibenz[b,e]Oxepine Derivative, 1-Hydroxy-10-Methoxy-Dibenz[b,e]Oxepin-6,11-Dione, from a Marine-Derived Fungus, *Beauveria Bassiana* TPU942. Mar. Drugs.

[54] Zhang J., Tao L., Liang Y., Chen L., Mi Y., Zheng L., Wang F., She Z., Lin Y., To K.K.W. (2010). Anthracenedione Derivatives as Anticancer Agents Isolated from Secondary Metabolites of the Mangrove Endophytic Fungi. Mar. Drugs.

[55] Tan Q.-W., Ouyang M.-A., Shen S., Li W. (2012). Bioactive Metabolites from a Marine-Derived Strain of the Fungus *Neosartorya Fischeri*. Nat. Prod. Res..

[56] Pang X., Cai G., Lin X., Salendra L., Zhou X., Yang B., Wang J., Wang J., Xu S., Liu Y. (2019). New Alkaloids and Polyketides from the Marine Sponge-Derived Fungus *Penicillium* Sp. SCSIO41015. Mar. Drugs.

[57] Chen Y., Cai X., Pan J., Gao J., Li J., Yuan J., Fu L., She Z., Lin Y. (2009). Structure Elucidation and NMR Assignments for Three Anthraquinone Derivatives from the Marine Fungus *Fusarium* Sp. (No. ZH-210). Magn. Reson Chem..

[58] Huang L., Zhang T., Li S., Duan J., Ye F., Li H., She Z., Gao G., Yang X. (2014). Anthraquinone G503 Induces Apoptosis in Gastric Cancer Cells through the Mitochondrial Pathway. PLoS ONE.

[59] Wu Z.-H., Liu D., Xu Y., Chen J.-L., Lin W.-H. (2018). Antioxidant Xanthones and Anthraquinones Isolated from a Marine-Derived Fungus *Aspergillus Versicolor*. Chin. J. Nat. Med..

[60] Wang C.C.C., Chiang Y.-M., Kuo P.-L., Chang J.-K., Hsu Y.-L. (2008). Norsolorinic Acid from Aspergillus Nidulans Inhibits the Proliferation of Human Breast Adenocarcinoma MCF-7 Cells via Fas-Mediated Pathway. Basic Clin. Pharmacol. Toxicol..

[61] Khamthong N., Rukachaisirikul V., Phongpaichit S., Preedanon S., Sakayaroj J. (2012). Bioactive Polyketides from the Sea Fan-Derived Fungus *Penicillium Citrinum* PSU-F51. Tetrahedron.

[62] Wijesekara I., Zhang C., Van Ta Q., Vo T.-S., Li Y.-X., Kim S.-K. (2014). Physcion from Marine-Derived Fungus *Microsporum* Sp. Induces Apoptosis in Human Cervical Carcinoma HeLa Cells. Microbiol. Res..

[63] Wang X., Tan T., Mao Z.-G., Lei N., Wang Z.-M., Hu B., Chen Z.-Y., She Z.-G., Zhu Y.-H., Wang H.-J. (2015). The Marine Metabolite SZ-685C Induces Apoptosis in Primary Human Nonfunctioning Pituitary Adenoma Cells by Ihibition of the Akt Pathway in Vitro. Mar. Drugs.

[64] Chen C.-H., Xiao W.-W., Jiang X.-B., Wang J.-W., Mao Z.-G., Lei N., Fan X., Song B.-B., Liao C.-X., Wang H.-J. (2013). A Novel Marine Drug, SZ-685C, Induces Apoptosis of MMQ Pituitary Tumor Cells by Downregulating MiR-200c. Curr. Med. Chem..

[65] Wang D., Wang S., Liu Q., Wang M., Wang C., Yang H. (2013). SZ-685C Exhibits Potent Anticancer Activity in Both Radiosensitive and Radioresistant NPC Cells through the MiR-205-PTEN-Akt Pathway. Oncol. Rep..

[66] Zhu X., He Z., Wu J., Yuan J., Wen W., Hu Y., Jiang Y., Lin C., Zhang Q., Lin M. (2012). A Marine Anthraquinone SZ-685C Overrides Adriamycin-Resistance in Breast Cancer Cells through Suppressing Akt Signaling. Mar. Drugs.

[67] Xie G., Zhu X., Li Q., Gu M., He Z., Wu J., Li J., Lin Y., Li M., She Z. (2010). SZ-685C, a Marine Anthraquinone, Is a Potent Inducer of Apoptosis with Anticancer Activity by Suppression of the Akt/FOXO Pathway: SZ-685C Induces Apoptosis and Inhibits Tumour Growth. Br. J. Pharmacol..

[68] Tuli H.S., Aggarwal V., Tuorkey M., Aggarwal D., Parashar N.C., Varol M., Savla R., Kaur G., Mittal S., Sak K. (2021). Emodin: A Metabolite That Exhibits Anti-Neoplastic Activities by Modulating Multiple Oncogenic Targets. Toxicol. In Vitro.

[69] Ye F., Chen C., Qin J., Liu J., Zheng C. (2015). Genetic Profiling Reveals an Alarming Rate of Cross-Contamination among Human Cell Lines Used in China. FASEB J..

[70] Bian X., Yang Z., Feng H., Sun H., Liu Y. (2017). A Combination of Species Identification and STR Profiling Identifies Cross-Contaminated Cells from 482 Human Tumor Cell Lines. Sci. Rep..

[71] Kamiloglu S., Sari G., Ozdal T., Capanoglu E. (2020). Guidelines for Cell Viability Assays. Food Front..

[72] D’Arcy M.S. (2019). Cell Death: A Review of the Major Forms of Apoptosis, Necrosis and Autophagy. Cell Biol. Int..

[73] Galluzzi L., Vitale I., Aaronson S.A., Abrams J.M., Adam D., Agostinis P., Alnemri E.S., Altucci L., Amelio I., Andrews D.W. (2018). Molecular Mechanisms of Cell Death: Recommendations of the Nomenclature Committee on Cell Death 2018. Cell Death Differ..

[74] Martini M., De Santis M.C., Braccini L., Gulluni F., Hirsch E. (2014). PI3K/AKT Signaling Pathway and Cancer: An Updated Review. Ann. Med..

[75] Yang J., Nie J., Ma X., Wei Y., Peng Y., Wei X. (2019). Targeting PI3K in Cancer: Mechanisms and Advances in Clinical Trials. Mol. Cancer.

[76] Greco G., Catanzaro E., Fimognari C. (2021). Natural Products as Inducers of Non-Canonical Cell Death: A Weapon against Cancer. Cancers.

[77] Zhou J., Li G., Han G., Feng S., Liu Y., Chen J., Liu C., Zhao L., Jin F. (2020). Emodin Induced Necroptosis in the Glioma Cell Line U251 via the TNF-α/RIP1/RIP3 Pathway. Invest. New Drugs.

[78] Si W., Shen J., Zheng H., Fan W. (2019). The Role and Mechanisms of Action of MicroRNAs in Cancer Drug Resistance. Clin. Epigenet.

[79] Li N., Wang C., Zhang P., You S. (2018). Emodin Inhibits Pancreatic Cancer EMT and Invasion by Up-regulating MicroRNA-1271. Mol. Med. Rep..

[80] Lin S.-Z., Xu J.-B., Ji X., Chen H., Xu H.-T., Hu P., Chen L., Guo J.-Q., Chen M.-Y., Lu D. (2015). Emodin Inhibits Angiogenesis in Pancreatic Cancer by Regulating the Transforming Growth Factor-β/Drosophila Mothers against Decapentaplegic Pathway and Angiogenesis-Associated MicroRNAs. Mol. Med. Rep..

[81] Lee H., Tsai S.-J. (1991). Effect of Emodin on Cooked-Food Mutagen Activation. Food Chem. Toxicol..

[82] Su H.-Y., Cherng S.-H., Chen C.-C., Lee H. (1995). Emodin Inhibits the Mutagenicity and DNA Adducts Induced by 1-Nitropyrene. Mutat. Res. Fundam. Mol. Mech. Mutagen..

[83] Wu C.H., Hsieh C.L., Song T.Y., Yen G.C. (2001). Inhibitory Effects of Cassia Tora L. on Benzo[a]Pyrene-Mediated DNA Damage toward HepG2 Cells. J. Agric. Food Chem..

[84] Słoczyńska K., Powroźnik B., Pękala E., Waszkielewicz A.M. (2014). Antimutagenic Compounds and Their Possible Mechanisms of Action. J. Appl. Genet..

[85] Bhattachar S. (2011). Natural Antimutagens: A Review. Res. J. Med. Plant..

[86] AbdelHakem A.M., Abdelhafez E.-S.M.N., Soloneski S.L., Larramendy M. (2021). Current trends and future perspectives of antimutagenic agents. Genotoxicity and Mutagenicity-Mechanisms and Test Methods.

[87] Sun M., Sakakibara H., Ashida H., Danno G., Kanazawa K. (2000). Cytochrome P4501A1-Inhibitory Action of Antimutagenic Anthraquinones in Medicinal Plants and the Structure-Activity Relationship. Biosci. Biotechnol. Biochem..

[88] Sevcovicova A., Bodnarova K., Loderer D., Imreova P., Galova E., Miadokova E. (2014). Dual Activities of Emodin—DNA Protectivity vs Mutagenicity. Neuro Endocrinol. Lett..

[89] Shah M.A., Adnan M., Rasul A., Hussain G., Sarfraz I., Nageen B., Riaz A., Khalid R., Asrar M., Selamoglu Z. (2020). Physcion and Physcion 8-O-β-D-Glucopyranoside: Natural Anthraquinones with Potential Anti-Cancer Activities. Curr. Drug Targets.

[90] Lippai M., Szatmári Z. (2017). Autophagy-from Molecular Mechanisms to Clinical Relevance. Cell Biol. Toxicol..

[91] Pang M.-J., Yang Z., Zhang X.-L., Liu Z.-F., Fan J., Zhang H.-Y. (2016). Physcion, a Naturally Occurring Anthraquinone Derivative, Induces Apoptosis and Autophagy in Human Nasopharyngeal Carcinoma. Acta Pharmacol. Sin..

[92] Aubrey B.J., Kelly G.L., Janic A., Herold M.J., Strasser A. (2018). How Does P53 Induce Apoptosis and How Does This Relate to P53-Mediated Tumour Suppression?. Cell Death Differ..

[93] Moreira T.F., Sorbo J.M., de Oliveira Souza F., Fernandes B.C., Ocampos F.M.M., de Oliveira Soares D.M., Arcaro C.A., Assis R.P., Barison A., Miguel O.G. (2018). Emodin, Physcion, and Crude Extract of *Rhamnus Sphaerosperma* var. Pubescens Induce Mixed Cell Death, Increase in Oxidative Stress, DNA Damage, and Inhibition of AKT in Cervical and Oral Squamous Carcinoma Cell Lines. Oxid Med. Cell Longev..

[94] Niu Y., Zhang J., Tong Y., Li J., Liu B. (2019). Physcion 8-O-β-Glucopyranoside Induced Ferroptosis via Regulating MiR-103a-3p/GLS2 Axis in Gastric Cancer. Life Sci..

[95] Durán N., Teixeira M.F.S., De Conti R., Esposito E. (2002). Ecological-Friendly Pigments from Fungi. Crit. Rev. Food Sci. Nutr..

[96] Mapari S.A., Nielsen K.F., Larsen T.O., Frisvad J.C., Meyer A.S., Thrane U. (2005). Exploring Fungal Biodiversity for the Production of Water-Soluble Pigments as Potential Natural Food Colorants. Curr. Opin. Biotechnol..

[97] Frisvad J.C., Filtenborg O. (1989). *Terverticillate Penicillia*: Chemotaxonomy and Mycotoxin Production. Mycologia.

[98] Mah L.-J., El-Osta A., Karagiannis T.C. (2010). GammaH2AX: A Sensitive Molecular Marker of DNA Damage and Repair. Leukemia.

[99] Solier S., Pommier Y. (2014). The Nuclear γ-H2AX Apoptotic Ring: Implications for Cancers and Autoimmune Diseases. Cell Mol. Life Sci..

[100] Chen W.-S., Hou J.-N., Guo Y.-B., Yang H.-L., Xie C.-M., Lin Y.-C., She Z.-G. (2011). Bostrycin Inhibits Proliferation of Human Lung Carcinoma A549 Cells via Downregulation of the PI3K/Akt Pathway. J. Exp. Clin. Cancer Res..

[101] Soleimani A., Rahmani F., Ferns G.A., Ryzhikov M., Avan A., Hassanian S.M. (2019). Role of Regulatory Oncogenic or Tumor Suppressor MiRNAs of PI3K/AKT Signaling Axis in the Pathogenesis of Colorectal Cancer. Curr. Pharm. Des..

[102] Gasparri M.L., Besharat Z.M., Farooqi A.A., Khalid S., Taghavi K., Besharat R.A., Sabato C., Papadia A., Panici P.B., Mueller M.D. (2018). MiRNAs and Their Interplay with PI3K/AKT/MTOR Pathway in Ovarian Cancer Cells: A Potential Role in Platinum Resistance. J. Cancer Res. Clin. Oncol..

[103] Rahmani F., Ziaeemehr A., Shahidsales S., Gharib M., Khazaei M., Ferns G.A., Ryzhikov M., Avan A., Hassanian S.M. (2020). Role of Regulatory MiRNAs of the PI3K/AKT/MTOR Signaling in the Pathogenesis of Hepatocellular Carcinoma. J. Cell Physiol..

[104] Kale J., Osterlund E.J., Andrews D.W. (2018). BCL-2 Family Proteins: Changing Partners in the Dance towards Death. Cell Death Differ..

[105] Pereira D.M., Valentão P., Correia-da-Silva G., Teixeira N., Andrade P.B. (2015). Translating Endoplasmic Reticulum Biology into the Clinic: A Role for ER-Targeted Natural Products?. Nat. Prod. Rep..

[106] Schirrmacher V. (2019). From Chemotherapy to Biological Therapy: A Review of Novel Concepts to Reduce the Side Effects of Systemic Cancer Treatment. Int. J. Oncol..

[107] Ferrari E., Gandellini P. (2020). Unveiling the Ups and Downs of MiR-205 in Physiology and Cancer: Transcriptional and Post-Transcriptional Mechanisms. Cell Death Dis..

[108] Li X.-H., Wang E.L., Zhou H.-M., Yoshimoto K., Qian Z.R. (2014). MicroRNAs in Human Pituitary Adenomas. Int. J. Endocrinol..

[109] Los M., Maddika S., Erb B., Schulze-Osthoff K. (2009). Switching Akt: From Survival Signaling to Deadly Response. Bioessays.

[110] Zhang X., Tang N., Hadden T.J., Rishi A.K. (2011). Akt, FoxO and Regulation of Apoptosis. Biochim. Biophys. Acta Mol. Cell Res..

[111] Kantari C., Walczak H. (2011). Caspase-8 and Bid: Caught in the Act between Death Receptors and Mitochondria. Biochim. Biophys. Acta Mol. Cell Res..

[112] Wright A., Nielson J., Tapiolas D., Motti C., Ovenden S.P., Kearns P., Liptrot C. (2009). Detailed NMR, Including 1,1-ADEQUATE, and Anticancer Studies of Compounds from the Echinoderm *Colobometra Perspinosa*. Mar. Drugs.

[113] Wätjen W., Ebada S.S., Bergermann A., Chovolou Y., Totzke F., Kubbutat M.H.G., Lin W., Proksch P. (2017). Cytotoxic Effects of the Anthraquinone Derivatives 1′-Deoxyrhodoptilometrin and (S)-(−)-Rhodoptilometrin Isolated from the Marine Echinoderm *Comanthus* sp.. Arch. Toxicol..

[114] Khokhar S., Pierens G.K., Hooper J.N.A., Ekins M.G., Feng Y., Davis R.A. (2016). Rhodocomatulin-Type Anthraquinones from the Australian Marine Invertebrates *Clathria Hirsuta* and *Comatula Rotalaria*. J. Nat. Prod..

[115] Huang Y.-F., Tian L., Fu H.-W., Hua H.-M., Pei Y.-H. (2006). One New Anthraquinone from Marine *Streptomyces* Sp. FX-58. Nat. Prod. Res..

[116] Zhang H., Wang H., Cui H., Li Z., Xie Z., Pu Y., Li F., Qin S. (2011). A New Anthracene Derivative from Marine *Streptomyces* Sp. W007 Exhibiting Highly and Selectively Cytotoxic Activities. Mar. Drugs.

[117] Lai Z., Yu J., Ling H., Song Y., Yuan J., Ju J., Tao Y., Huang H. (2018). Grincamycins I–K, Cytotoxic Angucycline Glycosides Derived from Marine-Derived Actinomycete *Streptomyces Lusitanus* SCSIO LR32. Planta Med..

[118] Song Y., Liu G., Li J., Huang H., Zhang X., Zhang H., Ju J. (2015). Cytotoxic and Antibacterial Angucycline- and Prodigiosin- Analogues from the Deep-Sea Derived *Streptomyces* Sp. SCSIO 11594. Mar. Drugs.

[119] Qu X.-Y., Ren J.-W., Peng A.-H., Lin S.-Q., Lu D.-D., Du Q.-Q., Liu L., Li X., Li E.-W., Xie W.-D. (2019). Cytotoxic, Anti-Migration, and Anti-Invasion Activities on Breast Cancer Cells of Angucycline Glycosides Isolated from a Marine-Derived *Streptomyces* sp.. Mar. Drugs.

[120] Peng A., Qu X., Liu F., Li X., Li E., Xie W. (2018). Angucycline Glycosides from an Intertidal Sediments Strain *Streptomyces* Sp. and Their Cytotoxic Activity against Hepatoma Carcinoma Cells. Mar. Drugs.

[121] Hu Y., Martinez E.D., MacMillan J.B. (2012). Anthraquinones from a Marine-Derived *Streptomyces Spinoverrucosus*. J. Nat. Prod..

[122] Huang H., Yang T., Ren X., Liu J., Song Y., Sun A., Ma J., Wang B., Zhang Y., Huang C. (2012). Cytotoxic Angucycline Class Glycosides from the Deep Sea Actinomycete *Streptomyces Lusitanus* SCSIO LR32. J. Nat. Prod..

[123] Zhu X., Duan Y., Cui Z., Wang Z., Li Z., Zhang Y., Ju J., Huang H. (2017). Cytotoxic Rearranged Angucycline Glycosides from Deep Sea-Derived *Streptomyces Lusitanus* SCSIO LR32. J. Antibiot..

[124] Xie Z., Liu B., Wang H., Yang S., Zhang H., Wang Y., Ji N., Qin S., Laatsch H. (2012). Kiamycin, a Unique Cytotoxic Angucyclinone Derivative from a Marine *Streptomyces* sp.. Mar. Drugs.

[125] Martin G.D.A., Tan L.T., Jensen P.R., Dimayuga R.E., Fairchild C.R., Raventos-Suarez C., Fenical W. (2007). Marmycins A and B, Cytotoxic Pentacyclic C-Glycosides from a Marine Sediment-Derived Actinomycete Related to the Genus *Streptomyces*. J. Nat. Prod..

[126] Adinarayana G., Venkateshan M.R., Bapiraju V.V.S.N.K., Sujatha P., Premkumar J., Ellaiah P., Zeeck A. (2006). Cytotoxic Compounds from the Marine Actinobacterium *Streptomyces Corchorusii* AUBN1/71. Russ. J. Bioorg. Chem..

[127] Murphy B.T., Narender T., Kauffman C.A., Woolery M., Jensen P.R., Fenical W. (2010). Saliniquinones A-F, New Members of the Highly Cytotoxic Anthraquinone-γ-Pyrones from the Marine Actinomycete *Salinispora Arenicola*. Aust. J. Chem..

[128] Lu Y., Xing Y., Chen C., Lu J., Ma Y., Xi T. (2012). Anthraquinone Glycosides from Marine *Streptomyces* Sp. Strain. Phytochem. Lett..

[129] Shrimali D., Shanmugam M.K., Kumar A.P., Zhang J., Tan B.K.H., Ahn K.S., Sethi G. (2013). Targeted Abrogation of Diverse Signal Transduction Cascades by Emodin for the Treatment of Inflammatory Disorders and Cancer. Cancer Lett..

[130] Wei W.-T., Lin S.-Z., Liu D.-L., Wang Z.-H. (2013). The Distinct Mechanisms of the Antitumor Activity of Emodin in Different Types of Cancer. Oncol. Rep..

[131] Abdelfattah M.S., Elmallah M.I.Y., Faraag A.H.I., Hebishy A.M.S., Ali N.H. (2018). Heliomycin and Tetracinomycin D: Anthraquinone Derivatives with Histone Deacetylase Inhibitory Activity from Marine Sponge-Associated *Streptomyces* Sp. SP9. 3 Biotech.

[132] Qiao X., Gan M., Wang C., Liu B., Shang Y., Li Y., Chen S. (2019). Tetracenomycin X Exerts Antitumour Activity in Lung Cancer Cells through the Downregulation of Cyclin D1. Mar. Drugs.

[133] Kelly A.D., Issa J.-P.J. (2017). The Promise of Epigenetic Therapy: Reprogramming the Cancer Epigenome. Curr. Opin. Genet. Dev..

[134] Peng X., Sun Z., Kuang P., Chen J. (2020). Recent Progress on HDAC Inhibitors with Dual Targeting Capabilities for Cancer Treatment. Eur. J. Med. Chem..

[135] Losson H., Schnekenburger M., Dicato M., Diederich M. (2016). Natural Compound Histone Deacetylase Inhibitors (HDACi): Synergy with Inflammatory Signaling Pathway Modulators and Clinical Applications in Cancer. Molecules.

[136] Liu Y., Salvador L.A., Byeon S., Ying Y., Kwan J.C., Law B.K., Hong J., Luesch H. (2010). Anticolon Cancer Activity of Largazole, a Marine-Derived Tunable Histone Deacetylase Inhibitor. J. Pharmacol. Exp. Ther..

[137] Sun J., Wang J., Wang X., Liu H., Zhang M., Liu Y.-C., Zhang C., Su Y., Shen Y., Guo Y. (2017). Marine-Derived Chromopeptide A, a Novel Class I HDAC Inhibitor, Suppresses Human Prostate Cancer Cell Proliferation and Migration. Acta Pharmacol. Sin..

[138] Rohr J., Thiericke R. (1992). Angucycline Group Antibiotics. Nat. Prod. Rep..

[139] Kharel M.K., Pahari P., Shepherd M.D., Tibrewal N., Nybo S.E., Shaaban K.A., Rohr J. (2012). Angucyclines: Biosynthesis, Mode-of-Action, New Natural Products, and Synthesis. Nat. Prod. Rep..

[140] Phillips D.H., Arlt V.M. (2009). Genotoxicity: Damage to DNA and Its Consequences. EXS.

[141] Kennedy S.R., Loeb L.A., Herr A.J. (2012). Somatic Mutations in Aging, Cancer and Neurodegeneration. Mech. Ageing Dev..

[142] Weakley S.M., Jiang J., Kougias P., Lin P.H., Yao Q., Brunicardi F.C., Gibbs R.A., Chen C. (2010). Role of Somatic Mutations in Vascular Disease Formation. Expert Rev. Mol. Diagn..

[143] Review 2012-REACH-Chemicals-Environment-European Commission. https://ec.europa.eu/environment/chemicals/reach/review_2012_en.htm.

[144] Younes M., Aggett P., Aguilar F., Crebelli R., Filipič M., Frutos M.J., Galtier P., Gott D., Gundert-Remy U., EFSA Panel on Food Additives and Nutrient Sources added to Food (ANS) (2018). Safety of Hydroxyanthracene Derivatives for Use in Food. EFSA J..

[145] Lombardi N., Bettiol A., Crescioli G., Maggini V., Gallo E., Sivelli F., Sofi F., Gensini G.F., Vannacci A., Firenzuoli F. (2020). Association between Anthraquinone Laxatives and Colorectal Cancer: Protocol for a Systematic Review and Meta-Analysis. Syst. Rev..

[146] Saito S.T., Silva G., Pungartnik C., Brendel M. (2012). Study of DNA-Emodin Interaction by FTIR and UV-Vis Spectroscopy. J. Photochem. Photobiol. B.

[147] Müller S.O., Eckert I., Lutz W.K., Stopper H. (1996). Genotoxicity of the Laxative Drug Components Emodin, Aloe-Emodin and Danthron in Mammalian Cells: Topoisomerase II Mediated?. Mutat. Res..

[148] Li Y., Luan Y., Qi X., Li M., Gong L., Xue X., Wu X., Wu Y., Chen M., Xing G. (2010). Emodin Triggers DNA Double-Strand Breaks by Stabilizing Topoisomerase II-DNA Cleavage Complexes and by Inhibiting ATP Hydrolysis of Topoisomerase II. Toxicol. Sci..

[149] Amacher D.E., de Serres F.J. (1984). The L5178Y/TK Gene Mutation Assay System. Chemical Mutagens.

[150] Chen Y.-Y., Chiang S.-Y., Lin J.-G., Yang J.-S., Ma Y.-S., Liao C.-L., Lai T.-Y., Tang N.-Y., Chung J.-G. (2010). Emodin, Aloe-Emodin and Rhein Induced DNA Damage and Inhibited DNA Repair Gene Expression in SCC-4 Human Tongue Cancer Cells. Anticancer Res..

[151] Tice R.R., Agurell E., Anderson D., Burlinson B., Hartmann A., Kobayashi H., Miyamae Y., Rojas E., Ryu J.C., Sasaki Y.F. (2000). Single Cell Gel/Comet Assay: Guidelines for in Vitro and in Vivo Genetic Toxicology Testing. Environ. Mol. Mutagen..

[152] Mueller S.O., Stopper H., Dekant W. (1998). Biotransformation of the Anthraquinones Emodin and Chrysophanol by Cytochrome P450 Enzymes. Bioactivation to Genotoxic Metabolites. Drug Metab. Dispos..

[153] Stark A.A., Townsend J.M., Wogan G.N., Demain A.L., Manmade A., Ghosh A.C. (1978). Mutagenicity and Antibacterial Activity of Mycotoxins Produced by *Penicillium Islandicum* Sopp and *Penicillium Rugulosum*. J. Environ. Pathol. Toxicol..

[154] van der Hoeven J.C. (1985). Occurrence and Detection of Natural Mutagens and Modifying Factors in Food Products. Princess Takamatsu Symp..

[155] Bruggeman I.M., van der Hoeven J.C.M. (1984). Lack of Activity of the Bacterial Mutagen Emodin in HGPRT and SCE Assay with V79 Chinese Hamster Cells. Mutat. Res. Genet. Toxicol..

[156] Kevekordes S., Spielberger J., Burghaus C.M., Birkenkamp P., Zietz B., Paufler P., Diez M., Bolten C., Dunkelberg H. (2001). Micronucleus Formation in Human Lymphocytes and in the Metabolically Competent Human Hepatoma Cell Line Hep-G2: Results with 15 Naturally Occurring Substances. Anticancer Res..

[157] Mengs U., Krumbiegel G., Völkner W. (1997). Lack of Emodin Genotoxicity in the Mouse Micronucleus Assay. Mutat. Res..

[158] Dong X., Fu J., Yin X., Cao S., Li X., Lin L., Huyiligeqi H., Ni J. (2016). Emodin: A Review of Its Pharmacology, Toxicity and Pharmacokinetics. Phytother. Res..

[159] Lewis D.F., Ioannides C., Parke D.V. (1996). COMPACT and Molecular Structure in Toxicity Assessment: A Prospective Evaluation of 30 Chemicals Currently Being Tested for Rodent Carcinogenicity by the NCI/NTP. Environ. Health Perspect..

[160] National Toxicology Program NTP (2001). Toxicology and Carcinogenesis Studies of EMODIN (CAS NO. 518-82-1) Feed Studies in F344/N Rats and B6C3F1 Mice. Natl. Toxicol. Program. Tech. Rep. Ser..

[161] (1990). Dantron (Chrysazin; 1,8-Dihydroxyanthraquinone). IARC. Monogr. Eval Carcinog. Risks Hum..

[162] Zhang Z., Fu J., Yao B., Zhang X., Zhao P., Zhou Z. (2011). In Vitro Genotoxicity of Danthron and Its Potential Mechanism. Mutat. Res..

[163] Krivobok S., Seigle-Murandi F., Steiman R., Marzin D.R., Betina V. (1992). Mutagenicity of Substituted Anthraquinones in the Ames/Salmonella Microsome System. Mutat. Res..

[164] Ryden E. (2000). Comparison of the Sensitivities of Salmonella Typhimurium Strains TA102 and TA2638A to 16 Mutagens. Mutagenesis.

[165] Simi S., Monrelli S., Gervasi P.G., Rainaldi G. (1995). Clastogenicity of Anthraquinones in V79 and in Three Derived Cell Lines Expressing P450 Enzymes. Mutat. Res. Lett..

[166] Kawai K., Mori H., Sugie S., Yoshimi N., Inoue T., Nakamaru T., Nozawa Y., Matsushima T. (1986). Genotoxicity in the Hepatocyte/DNA Repair Test and Toxicity to Liver Mitochondria of 1-Hydroxyanthraquinone and Several Dihydroxyanthraquinones. Cell Biol. Toxicol..

[167] Mueller S.O., Stopper H. (1999). Characterization of the Genotoxicity of Anthraquinones in Mammalian Cells. Biochim. Biophys. Acta.

[168] Mori H., Sugie S., Niwa K., Takahashi M., Kawai K. (1985). Induction of Intestinal Tumours in Rats by Chrysazin. Br. J. Cancer.

[169] Mori H., Sugie S., Niwa K., Yoshimi N., Tanaka T., Hirono I. (1986). Carcinogenicity of Chrysazin in Large Intestine and Liver of Mice. Jpn. J. Cancer Res..

[170] (2012). EFSA Scientific Committee Guidance on Selected Default Values to Be Used by the EFSA Scientific Committee, Scientific Panels and Units in the Absence of Actual Measured Data. EFSA J..

[171] Xie L., Tang H., Song J., Long J., Zhang L., Li X. (2019). Chrysophanol: A Review of Its Pharmacology, Toxicity and Pharmacokinetics. J. Pharm Pharmacol..

[172] Yang X.M., Li J.S., Huang G.X., Li Q.Q., Yan L.J. (2012). Study on Potential Toxic Mechanism of Chrysophanol Binding DNA by Saturation Value Binding DNA. Asian J. Chem..

[173] Yang X.M., Li J.S., Li Q.Q., Huang G.X., Yan L.J. (2011). Evaluation of the Potential Toxicity of Anthraquinone Derivatives in Chinese Herbal Medicines by the Resonance Light Scattering Spectrum. Asian J. Chem..

[174] Tikkanen L., Matsushima T., Natori S. (1983). Mutagenicity of Anthraquinones in the Salmonella Preincubation Test. Mutat. Res..

[175] Mengs U., Schuler D., Marshall R.R. (2001). No Induction of Chromosomal Aberrations in Chinese Hamster Ovary Cells by Chrysophanol. Mutat. Res..

[176] Heidemann A., Völkner W., Mengs U. (1996). Genotoxicity of Aloeemodin in Vitro and in Vivo. Mutat. Res..

[177] Özenver N., Saeed M., Demirezer L.Ö., Efferth T. (2018). Aloe-Emodin as Drug Candidate for Cancer Therapy. Oncotarget.

[178] Nesslany F., Simar-Meintières S., Ficheux H., Marzin D. (2009). Aloe-Emodin-Induced DNA Fragmentation in the Mouse in Vivo Comet Assay. Mutat. Res..

[179] Yu C.-P., Shia C.-S., Lin H.-J., Hsieh Y.-W., Lin S.-P., Hou Y.-C. (2016). Analysis of the Pharmacokinetics and Metabolism of Aloe-Emodin Following Intravenous and Oral Administrations in Rats: Pharmacokinetics and Metabolism of Aloe-Emodin. Biomed. Chromatogr..

[180] Dong X., Zeng Y., Liu Y., You L., Yin X., Fu J., Ni J. (2020). Aloe-emodin: A Review of Its Pharmacology, Toxicity, and Pharmacokinetics. Phytother Res..

[181] Westendorf J., Marquardt B., Poginski M., Dominiak J., Schmidt H., Marquardt H. (1990). Genotoxicity of Naturally Occurring Hydroxyanthraquinones. Mutat. Res..

[182] Mengs U., Heidemann A. (1993). Genotoxicity of Sennosides and Rhein in Vitro and in Vivo. Med. Sci..

[183] Nohmi T. (2018). Thresholds of Genotoxic and Non-Genotoxic Carcinogens. Toxicol. Res..

[184] Chen C., Gao J., Wang T.-S., Guo C., Yan Y.-J., Mao C.-Y., Gu L.-W., Yang Y., Li Z.-F., Liu A. (2018). NMR-Based Metabolomic Techniques Identify the Toxicity of Emodin in HepG2 Cells. Sci. Rep..

[185] Anthraquinone-Registration Dossier-ECHA. https://echa.europa.eu/registration-dossier/-/registered-dossier/5769/7/7/3.

[186] EUR-Lex-Ares(2020)1357432-EN-EUR-Lex. https://eur-lex.europa.eu/legal-content/EN/TXT/?uri=pi_com%3AAres%282020%291357432.

